# Supplementary material for: Invasive vegetation affects amphibian skin microbiota and body condition
Source: PeerJ. 2020 Feb 19;8:e8549. doi: 10.7717/peerj.8549 (PMC7035873; doi:10.7717/peerj.8549)
Supplement: Supplemental Information 1 [file peerj-08-8549-s001.zip › PeerJ Supp/TableS1.pdf]

		LDA stats		*Eucalyptus* Sites			*Quercus* sites			Taxonomy						
Assignment	ASV ID	LDA	p.value	E1	E2	E3	N1	N2	N3	Kingdom	Phylum	Class	Order	Family	Genus	Species
Eucalyptus	62ab3ec11e2c7d247ec90a010f08b127	0.56	0.025	0	1.3E-03	3.4E-04	0	7.3E-05	0	Archaea	Crenarchaeota	Thaumarchaeota	Nitrososphaerales	Nitrososphaeraceae	*Candidatus Nitrososphaera*	
	1fad3b361787345c5c1012dbca74b747	0.52	0.035	0	9.8E-04	5.6E-04	0	0	0	Bacteria	Acidobacteria	Chloracidobacteria	RB41	Ellin6075		
	6424250710a6c8f3ecd0fd26603bcbdb	0.52	0.04	3.9E-04	0	3.2E-04	0	0	0	Bacteria	Acidobacteria	Chloracidobacteria	RB41	Ellin6075		
	959dc33fb13c310dd52835caa52eeca4	0.81	0.005	5.4E-04	6.7E-03	1.0E-02	5.4E-04	4.9E-05	0	Bacteria	Acidobacteria	Chloracidobacteria	RB41			
	4066eea496dc43011db89efa755d95ba	0.52	0.04	0	9.1E-04	1.5E-03	0	0	0	Bacteria	Acidobacteria	Chloracidobacteria	RB41			
	20b686910cf9db4b08f7b7c220800316	0.73	0.005	1.9E-03	7.3E-03	3.7E-03	1.0E-03	1.6E-03	0	Bacteria	Acidobacteria	Acidobacteria-6	iii1-15			
	83358a5ba1eb93b5fb9b0c26f03cfa16	0.63	0.005	2.2E-03	7.2E-04	3.4E-04	0	0	0	Bacteria	Acidobacteria	Acidobacteria-6	iii1-15			
	1c243c85d0ef1c7fb852db2ff20274c9	0.68	0.01	1.2E-03	1.3E-03	1.9E-03	0	0	0	Bacteria	Acidobacteria	Acidobacteria-6	iii1-15			
	4b6fedd1a8a1398c3536613393c715da	0.68	0.01	2.3E-03	2.5E-03	1.3E-03	0	0	0	Bacteria	Acidobacteria	Acidobacteria-6	iii1-15			
	d60f5b0e9d8b7216feda265f51c8cbfa	0.62	0.015	1.1E-03	0	1.1E-03	0	4.9E-04	3.5E-04	Bacteria	Acidobacteria	Acidobacteria-6	iii1-15			
	9be6b9155e116fa92bdea784765acef7	0.52	0.04	0	3.2E-03	2.2E-03	0	0	0	Bacteria	Acidobacteria	Acidobacteria-6	iii1-15			
	01e621899ab9f3a202c6c89a2f8dff7d	0.52	0.05	0	0	9.3E-04	0	0	0	Bacteria	Acidobacteria	Acidobacteriia	Acidobacteriales	Koribacteraceae	*Candidatus Koribacter*	
	be1265dcf110303ae9205c70df189f2c	0.58	0.02	0	1.9E-03	1.0E-03	0	0	0	Bacteria	Acidobacteria	Acidobacteriia	Acidobacteriales	Koribacteraceae		
	c76ccc5561c0d0afb24d0908d621e68b	0.58	0.02	0	3.3E-04	1.5E-03	0	0	0	Bacteria	Acidobacteria	Acidobacteriia	Acidobacteriales	Koribacteraceae		
	93639a4d6b4e945d9ac2d5b2de92b43c	0.52	0.035	0	1.8E-03	1.2E-03	0	0	0	Bacteria	Acidobacteria	Acidobacteriia	Acidobacteriales	Koribacteraceae		
	29867c223ffd53537c126ea6d169a81a	0.52	0.03	1.5E-03	0	1.7E-04	0	0	0	Bacteria	Acidobacteria	Solibacteres	Solibacterales	Bryobacteraceae		
	f735e0fdc4a05fa27bf9d42dfdf62d37	0.63	0.01	2.9E-04	3.3E-04	1.1E-03	0	0	0	Bacteria	Acidobacteria	Solibacteres	Solibacterales			
	08c9926357a5630b9690ef5d0d1ba37b	0.52	0.03	9.3E-04	0	6.4E-04	0	0	0	Bacteria	Acidobacteria	Sva0725	Sva0725			
	15d0c75db0d4705fd5dd4770a59c8f00	0.86	0.005	8.8E-04	5.2E-04	1.3E-03	0	0	0	Bacteria	Actinobacteria	Acidimicrobiia	Acidimicrobiales	EB1017		
	4ca1b0bd56684db2b0f71bb339bf8cd2	0.71	0.015	1.2E-03	2.0E-03	6.1E-04	5.9E-04	1.7E-04	0	Bacteria	Actinobacteria	Acidimicrobiia	Acidimicrobiales			
	20e46057c8224b4162d2c785d9bce76d	0.58	0.02	0	6.5E-05	3.9E-04	0	0	0	Bacteria	Actinobacteria	Acidimicrobiia	Acidimicrobiales			
	805e633f669e5e954d57037440aa8f67	0.57	0.05	2.0E-04	3.3E-04	3.0E-03	1.5E-04	0	0	Bacteria	Actinobacteria	Actinobacteria	Actinomycetales	Micrococcaceae	*Arthrobacter*	
	ee4cd11ee5afca311dbfd7b16c4002d6	0.63	0.015	1.4E-03	2.2E-03	1.7E-03	0	0	0	Bacteria	Actinobacteria	Actinobacteria	Actinomycetales	Micromonosporaceae		
	24f128b8910f158454543016c005181b	0.58	0.03	9.3E-04	0	8.1E-04	0	0	0	Bacteria	Actinobacteria	Actinobacteria	Actinomycetales	Nocardioidaceae	*Kribbella*	
	80d4a87f74628dfc2e7d82ffa9af449d	0.52	0.04	2.9E-04	7.2E-04	1.5E-04	0	0	0	Bacteria	Actinobacteria	Actinobacteria	Actinomycetales	Nocardioidaceae	*Kribbella*	
	113d585b3587ce9c265ffdf028af017d	0.52	0.04	0	0	4.2E-04	0	0	0	Bacteria	Actinobacteria	Actinobacteria	Actinomycetales	Promicromonosporaceae	*Promicromonospora*	
	286264c38ab5891097a4cdd5458a8769	0.52	0.05	3.4E-04	1.3E-03	8.8E-04	0	0	0	Bacteria	Actinobacteria	Actinobacteria	Actinomycetales	Pseudonocardiaceae	*Amycolatopsis*	
	6d80e3698883ee1b292e9477ff396ae0	0.57	0.025	0	2.0E-03	3.3E-03	0	0	7.8E-05	Bacteria	Actinobacteria	Actinobacteria	Actinomycetales	Streptomycetaceae	*Streptomyces*	*lanatus*
	832ccb367d0785dc8d1f9a2c75160d59	0.76	0.02	4.1E-03	4.6E-03	4.9E-03	0	4.2E-04	1.6E-03	Bacteria	Actinobacteria	Actinobacteria	Actinomycetales	Streptomycetaceae	*Streptomyces*	
	19bb331bff045e1a15cf99b8e35450cf	0.55	0.035	8.8E-04	1.4E-03	5.1E-04	0	1.7E-04	0	Bacteria	Actinobacteria	Actinobacteria	Actinomycetales			
	5ba570f4eb8bd01e7f620837413b7cce	0.52	0.045	2.4E-04	8.5E-04	2.2E-04	0	0	0	Bacteria	Actinobacteria	Actinobacteria	Actinomycetales			
	da4a2cf4c1b7ccac1f416243de701f04	0.52	0.05	0	0	2.7E-04	0	0	0	Bacteria	Actinobacteria	Actinobacteria	Micrococcales			
	a3f3934a2ac396a9db1469b32c3003db	0.52	0.035	0	6.5E-04	4.9E-04	0	0	0	Bacteria	Actinobacteria	Thermoleophilia	Gaiellales	Gaiellaceae		
	9cd340c16b0820cae84514e3820f91c9	0.58	0.03	0	1.2E-03	5.9E-04	0	0	0	Bacteria	Actinobacteria	Thermoleophilia	Solirubrobacterales	Patulibacteraceae		
	7f983d57978555310aca32018752e45b	0.58	0.03	0	2.6E-04	6.8E-04	0	0	0	Bacteria	Actinobacteria	Thermoleophilia	Solirubrobacterales			
	34fa12dfb373a1e0ed85ac3b648e85ed	0.52	0.04	5.9E-04	0	2.9E-04	0	0	0	Bacteria	Bacteroidetes	Saprospirae	Saprospirales	Chitinophagaceae	*Chitinophaga*	
	cce6af475addf6d911b2d8c7a9a7a534	0.60	0.02	1.4E-03	2.0E-04	1.5E-04	2.0E-04	0	0	Bacteria	Bacteroidetes	Saprospirae	Saprospirales	Chitinophagaceae	*Niastella*	
	2c019ec69d3c9041bb4c1c88817ebd23	0.58	0.02	0	1.1E-03	7.6E-04	0	0	0	Bacteria	Bacteroidetes	Saprospirae	Saprospirales	Chitinophagaceae		
	d71fa1946202725ad826186d6bc0dc9c	0.52	0.035	0	1.3E-04	4.9E-04	0	0	0	Bacteria	Bacteroidetes	Saprospirae	Saprospirales	Chitinophagaceae		
	a117969a926bdc003f6f144b9c94db95	0.52	0.04	3.9E-04	3.3E-04	3.2E-04	0	0	0	Bacteria	Bacteroidetes	Saprospirae	Saprospirales	Chitinophagaceae		
	3d34eca67e97b943c8d4f7a90e493b6e	0.67	0.005	7.8E-04	1.7E-03	1.5E-03	0	0	2.0E-04	Bacteria	Bacteroidetes	Cytophagia	Cytophagales	Cytophagaceae		
	15ad768dcbed591ede5271d32e1c493e	0.63	0.04	7.8E-04	3.9E-04	9.3E-04	0	1.7E-04	2.3E-04	Bacteria	Bacteroidetes	Cytophagia	Cytophagales	Cytophagaceae		
	38cf7ab9612a05fae726fcf961a1fd7c	0.57	0.04	4.7E-03	2.0E-03	5.6E-04	2.0E-04	0	0	Bacteria	Bacteroidetes	Cytophagia	Cytophagales	Cytophagaceae		
	6b94c1e0414c0e959c0a02bedb84c4db	0.60	0.05	6.4E-04	4.6E-04	8.6E-04	0	9.8E-05	1.6E-04	Bacteria	Bacteroidetes	Cytophagia	Cytophagales	Cytophagaceae		
	dfb6dd578dccfd219ef326b04ab4d1d3	0.58	0.03	4.9E-05	5.9E-04	5.9E-04	0	0	0	Bacteria	Chloroflexi	TK10				
	95fd02bf64a31ed3b9f31a01f2196b30	0.63	0.01	2.0E-04	0	1.4E-03	0	0	0	Bacteria	Gemmatimonadetes	Gemm-1				
	28059678966c19fe81314807f64e4a59	0.58	0.02	0	3.3E-04	3.9E-04	0	0	0	Bacteria	Gemmatimonadetes	Gemmatimonadetes	Ellin5290			
	ece4589880c25b4eab07b7890ccf05ec	0.52	0.045	4.9E-04	0	5.1E-04	0	0	0	Bacteria	Gemmatimonadetes	Gemmatimonadetes	Ellin5290			
	a8060846c26ec8ea3f9c22969ede199c	0.63	0.015	9.8E-04	3.3E-03	6.1E-04	0	0	0	Bacteria	Nitrospirae	Nitrospira	Nitrospirales	Nitrospiraceae	*Nitrospira*	
	996e6b9a589b70947dbc2ffa23d54c59	0.52	0.05	0	7.8E-04	5.9E-04	0	0	0	Bacteria	Nitrospirae	Nitrospira	Nitrospirales	Nitrospiraceae	*Nitrospira*	
	88bf884a2fc3a396f1f27016c435fcb1	0.52	0.05	0	0	6.1E-04	0	0	0	Bacteria	Planctomycetes	Phycisphaerae	WD2101			
	ef9bb014f26e380f0ce146da8fad3ed4	0.62	0.015	6.4E-04	6.5E-04	4.4E-04	0	4.9E-05	0	Bacteria	Planctomycetes	Planctomycetia	Gemmatales	Isosphaeraceae	*Singulisphaera*	
	190321d43c2176dbbd983d860a3c3616	0.58	0.01	0	0	5.6E-04	0	0	0	Bacteria	Planctomycetes	Planctomycetia	Pirellulales	Pirellulaceae		
	41d345aa787b2c4404488b1703611b72	0.63	0.005	1.2E-03	5.1E-03	6.6E-04	0	0	0	Bacteria	Proteobacteria	Alphaproteobacteria	Ellin329			
	078c73c2b9bfbac9f087fcdc4844e467	0.50	0.045	1.1E-03	0	1.6E-03	0	1.7E-04	0	Bacteria	Proteobacteria	Alphaproteobacteria	Rhizobiales	Bradyrhizobiaceae	*Bradyrhizobium*	
	33fd454253b9038b04dc90dc9902d0dc	0.68	0.015	1.3E-03	2.2E-03	8.6E-04	0	2.4E-05	0	Bacteria	Proteobacteria	Alphaproteobacteria	Rhizobiales	Hyphomicrobiaceae	*Pedomicrobium*	
	6500567dc89527d572e6fa3cdb246f9e	0.52	0.03	2.2E-03	0	2.1E-03	0	0	0	Bacteria	Proteobacteria	Alphaproteobacteria	Rhizobiales	Hyphomicrobiaceae	*Rhodoplanes*	
	bebaf5196b8c43f72b5b3dcf2adc8c8a	0.52	0.045	1.7E-03	2.0E-03	2.1E-03	0	0	0	Bacteria	Proteobacteria	Alphaproteobacteria	Rhizobiales	Hyphomicrobiaceae	*Rhodoplanes*	
	ac0cc4c4caa63978fcb9b60772a73825	0.57	0.05	1.9E-03	0	2.0E-03	7.3E-04	0	7.0E-04	Bacteria	Proteobacteria	Alphaproteobacteria	Rhizobiales	Hyphomicrobiaceae	*Rhodoplanes*	
	b5afd0d8966592de349a9805c386ee36	0.74	0.005	6.8E-04	2.6E-04	1.1E-03	1.5E-04	0	1.2E-04	Bacteria	Proteobacteria	Alphaproteobacteria	Rhizobiales	Rhodobiaceae	*Afifella*	
	ac68934aafcff691eedf9296809fd4b1	0.79	0.005	5.9E-04	3.3E-04	1.5E-03	2.9E-04	0	0	Bacteria	Proteobacteria	Alphaproteobacteria	Rhizobiales			
	540bd048de0e54693e7e4655c4196ec0	0.52	0.025	7.8E-04	0	3.4E-04	0	0	0	Bacteria	Proteobacteria	Alphaproteobacteria	Rhizobiales			
	1636dec245e9d17c45d4f0cd1db2de35	0.58	0.02	1.6E-03	3.3E-04	2.0E-04	0	0	0	Bacteria	Proteobacteria	Alphaproteobacteria	Rhodobacterales	Hyphomonadaceae		
	6ef3d9b6417d9ed7236f82a1b236382e	0.52	0.025	0	2.0E-04	2.2E-04	0	0	0	Bacteria	Proteobacteria	Alphaproteobacteria	Rhodobacterales	Hyphomonadaceae		
	6dfaef1a993a422533761a3936f1aa08	0.62	0.03	5.9E-04	1.3E-03	6.1E-04	0	3.4E-04	0	Bacteria	Proteobacteria	Alphaproteobacteria	Rhodobacterales	Hyphomonadaceae		
	cb38fa4204b8f078ded9a296b14549f6	0.84	0.005	1.9E-03	5.1E-03	5.9E-03	2.0E-03	7.6E-04	7.8E-04	Bacteria	Proteobacteria	Alphaproteobacteria	Rhodospirillales	Rhodospirillaceae	*Reyranella*	*massiliensis*
	822754e7f9add2d17f33255c21748e29	0.89	0.005	7.3E-04	1.0E-03	8.6E-04	0	0	2.7E-04	Bacteria	Proteobacteria	Alphaproteobacteria	Rhodospirillales	Rhodospirillaceae		
	243bc6b6c2535fa71c5860cd5489d4bc	0.67	0.01	8.3E-04	6.5E-04	3.9E-04	9.8E-05	0	0	Bacteria	Proteobacteria	Alphaproteobacteria	Rhodospirillales	Rhodospirillaceae		
	3c3a2e828123e5cd4f884045aae6f234	0.68	0.01	2.9E-04	2.0E-04	6.1E-04	0	0	0	Bacteria	Proteobacteria	Alphaproteobacteria	Rhodospirillales	Rhodospirillaceae		
	f2139fee20c8ee91ca62180de29b60b2	0.68	0.01	1.7E-03	6.5E-04	2.9E-03	0	0	0	Bacteria	Proteobacteria	Alphaproteobacteria	Rhodospirillales	Rhodospirillaceae		
	047c3fe0b53609f1bbe562984062420e	0.58	0.03	0	0	2.1E-03	0	0	0	Bacteria	Proteobacteria	Alphaproteobacteria	Rhodospirillales	Rhodospirillaceae		
	346f6286f57bc3152e02f87c44386528	0.62	0.035	6.8E-04	9.1E-04	3.3E-03	5.4E-04	4.9E-04	3.1E-04	Bacteria	Proteobacteria	Alphaproteobacteria	Rhodospirillales	Rhodospirillaceae		
	dd3c64275e428393919575f2ebf04f9a	0.52	0.035	0	9.1E-04	1.7E-04	0	0	0	Bacteria	Proteobacteria	Alphaproteobacteria	Rhodospirillales	Rhodospirillaceae		
	c57b681ed4a14f8be0558a68a7c2f8d5	0.57	0.045	2.0E-04	3.3E-04	1.4E-03	7.8E-04	0	0	Bacteria	Proteobacteria	Alphaproteobacteria	Rhodospirillales	Rhodospirillaceae		
	6596f231b09d1cdc975931169bdebfef	0.52	0.05	2.0E-04	0	5.9E-04	0	0	0	Bacteria	Proteobacteria	Alphaproteobacteria	Rhodospirillales	Rhodospirillaceae		
	8b5de61b51fee6d382de99cffeca85bf	0.63	0.03	2.0E-03	1.4E-03	1.5E-03	0	5.9E-04	0	Bacteria	Proteobacteria	Alphaproteobacteria	Sphingomonadales	Sphingomonadaceae	*Kaistobacter*	
	26dced51f936f6ac6f6200bd49079161	0.82	0.005	4.0E-03	3.9E-04	1.5E-03	0	0	0	Bacteria	Proteobacteria	Alphaproteobacteria	Sphingomonadales	Sphingomonadaceae	*Novosphingobium*	
	9c0f13da7e122b66fbc8fedd932c6214	0.59	0.01	1.7E-03	0	8.3E-04	5.4E-04	0	0	Bacteria	Proteobacteria	Alphaproteobacteria	Sphingomonadales	Sphingomonadaceae	*Sphingomonas*	*wittichii*
	ad759d9fbfce393e4857716f753d9bef	0.60	0.015	3.6E-03	0	2.1E-03	0	4.2E-04	0	Bacteria	Proteobacteria	Alphaproteobacteria	Sphingomonadales	Sphingomonadaceae	*Sphingomonas*	
	ca9b619e409b1724291a4ac74f9eabf8	0.63	0.005	1.7E-03	4.6E-04	6.8E-04	0	0	0	Bacteria	Proteobacteria	Alphaproteobacteria				
	e007858c3abd7a15cf8e8930820a689e	0.73	0.005	2.1E-03	1.1E-03	1.4E-03	0	0	0	Bacteria	Proteobacteria	Alphaproteobacteria				
	54b522d661ef4ef31d328e63de4392e3	0.73	0.01	1.1E-03	2.6E-03	1.4E-03	3.4E-04	2.4E-05	4.3E-04	Bacteria	Proteobacteria	Alphaproteobacteria				
	dcb94256a65a73793393f0f79e3340ed	0.68	0.01	8.8E-04	7.2E-04	4.6E-04	0	0	0	Bacteria	Proteobacteria	Alphaproteobacteria				
	881e83221791aa24a0085fa22dab4ac7	0.52	0.035	7.8E-04	0	1.4E-03	0	0	0	Bacteria	Proteobacteria	Betaproteobacteria	Burkholderiales	Comamonadaceae		
	b79add6bd2c9329ce98744210edfabe2	0.45	0.05	0	0	7.6E-04	0	0	0	Bacteria	Proteobacteria	Betaproteobacteria	Burkholderiales	Oxalobacteraceae	*Massilia*	*dura*
	5e91c08749688980ba118988c5e0b879	0.57	0.04	3.5E-03	0	1.8E-03	2.4E-04	0	0	Bacteria	Proteobacteria	Betaproteobacteria	Ellin6067			
	999e7806b23a27b7759c33ac170411f7	0.76	0.005	3.3E-03	2.6E-03	3.4E-03	3.4E-04	0	3.9E-05	Bacteria	Proteobacteria	Betaproteobacteria	IS-44			
	685c3f01d0150013e91f92ad0647c28e	0.61	0.04	2.8E-03	2.5E-03	2.5E-03	0	1.3E-03	0	Bacteria	Proteobacteria	Betaproteobacteria	IS-44			
	f5a0f8706517672628fb62471c7f3415	0.73	0.005	1.9E-03	2.6E-03	1.2E-03	8.3E-04	0	0	Bacteria	Proteobacteria	Betaproteobacteria	MND1			
	92434fc13ec4c8dcf05a665a92645f51	0.57	0.025	1.1E-03	4.6E-04	4.2E-04	0	4.9E-05	0	Bacteria	Proteobacteria	Betaproteobacteria	MND1			
	8f11af15abdf5815cc0c0b504250191a	0.63	0.005	3.4E-04	4.6E-04	1.9E-03	0	0	0	Bacteria	Proteobacteria	Betaproteobacteria	Nitrosomonadales	Nitrosomonadaceae	*Nitrosovibrio*	*tenuis*
	ae1296a72210ed715126fa22557b2616	0.58	0.02	0	2.3E-03	3.1E-03	0	0	0	Bacteria	Proteobacteria	Betaproteobacteria	SC-I-84			
	978676c771c403130460e1e004347be3	0.63	0.005	1.4E-03	2.3E-03	3.7E-04	0	0	0	Bacteria	Proteobacteria	Betaproteobacteria				
	f3fa5ce15414840a15c0ecb93c1916f4	0.63	0.015	7.8E-04	3.5E-03	1.4E-03	0	0	0	Bacteria	Proteobacteria	Betaproteobacteria				
	557aef6a368d8d210629831c1a366d82	0.50	0.05	1.6E-03	0	5.1E-04	0	9.8E-05	0	Bacteria	Proteobacteria	Deltaproteobacteria	Myxococcales	Haliangiaceae		
	e0bd1633b7ef2d298dae21f0d631571b	0.52	0.05	7.3E-04	4.1E-03	2.9E-04	0	0	0	Bacteria	Proteobacteria	Deltaproteobacteria	Myxococcales	Haliangiaceae		
	7a68de5cb85f1a1c69094d2bcc4652ca	0.79	0.005	1.3E-03	3.3E-04	9.8E-04	0	0	5.9E-04	Bacteria	Proteobacteria	Deltaproteobacteria	Myxococcales	Polyangiaceae		
	2511ff5d78ef6d53e9819a09ca5d9eea	0.67	0.005	2.6E-03	5.2E-04	2.7E-04	0	0	1.2E-04	Bacteria	Proteobacteria	Deltaproteobacteria	Myxococcales			
	1e17ca77fd31f27a32608cc77d0a4f5d	0.58	0.015	2.0E-04	2.6E-04	2.9E-04	0	0	0	Bacteria	Proteobacteria	Deltaproteobacteria	Myxococcales			
	87a87c4f8100e4f6e1d8904ba73145bf	0.63	0.015	9.8E-04	9.1E-04	9.0E-04	0	0	0	Bacteria	Proteobacteria	Deltaproteobacteria	Myxococcales			
	a0bd9ed383c2ea78c55780e41d590714	0.58	0.02	0	0	9.5E-04	0	0	0	Bacteria	Proteobacteria	Deltaproteobacteria	Myxococcales			
	f6720b937fab5a8659272ad598284f64	0.52	0.03	7.3E-04	0	5.6E-04	0	0	0	Bacteria	Proteobacteria	Deltaproteobacteria	Myxococcales			
	4324014bb414626d0f3ec39c5cc86d92	0.52	0.035	3.9E-04	3.8E-03	4.4E-04	0	0	0	Bacteria	Proteobacteria	Deltaproteobacteria	Myxococcales			
	2873f6cace80f4bdc21f4b1b7fd6e05d	0.61	0.025	2.9E-03	2.4E-03	3.7E-04	0	2.7E-04	0	Bacteria	Proteobacteria	Deltaproteobacteria	Syntrophobacterales	Syntrophobacteraceae		
	34d1053233c65c82c1aba56a3bc3040a	0.58	0.03	0	3.3E-04	5.6E-04	0	0	0	Bacteria	Proteobacteria	Deltaproteobacteria	Syntrophobacterales	Syntrophobacteraceae		
	6d05de90587e0bff672b63e58c28c0ab	0.52	0.035	0	2.0E-04	4.4E-04	0	0	0	Bacteria	Proteobacteria	Gammaproteobacteria	Alteromonadales	OM60		

	52ef4e5fd1f06bd01003c5f0591376b1	0.68	0.005	7.3E-04	5.3E-03	7.6E-04	0	0	0	Bacteria	Proteobacteria	Gammaproteobacteria	Thiotrichales	Piscirickettsiaceae		
	40556136a0a6807e97d5d4393cd79a3c	0.84	0.005	4.9E-03	1.5E-02	6.1E-03	1.3E-03	1.4E-03	0	Bacteria	Proteobacteria	Gammaproteobacteria	Xanthomonadales	Sinobacteraceae	*Steroidobacter*	
	a3b2563c2e0b9dc498975725b75fc560	0.82	0.005	2.7E-03	8.3E-03	2.6E-03	0	0	0	Bacteria	Proteobacteria	Gammaproteobacteria	Xanthomonadales	Sinobacteraceae	*Steroidobacter*	
	c164fd002b5502c5963286a9322833a0	0.96	0.005	4.8E-03	6.4E-03	6.2E-03	0	1.2E-04	0	Bacteria	Proteobacteria	Gammaproteobacteria	Xanthomonadales	Sinobacteraceae	*Steroidobacter*	
	df3b0b6d184909a507be2c2edacc0d1c	0.73	0.005	1.1E-03	7.8E-04	9.8E-04	0	0	0	Bacteria	Proteobacteria	Gammaproteobacteria	Xanthomonadales	Sinobacteraceae	*Steroidobacter*	
	da9ae48df4bbb540a1586c06e84aae17	0.58	0.02	5.9E-04	1.6E-03	1.9E-03	0	0	0	Bacteria	Proteobacteria	Gammaproteobacteria	Xanthomonadales	Sinobacteraceae	*Steroidobacter*	
	e98349f8b3c7732e4c4148cab1686545	0.74	0.005	3.3E-03	6.1E-03	4.1E-03	8.3E-04	3.9E-04	0	Bacteria	Proteobacteria	Gammaproteobacteria	Xanthomonadales	Sinobacteraceae		
	df250b4a5a282758ae2885d854c3c4c2	0.52	0.045	0	0	1.2E-03	0	0	0	Bacteria	Proteobacteria	Gammaproteobacteria	Xanthomonadales	Sinobacteraceae		
	754cde8e37519fa90d455c14e227a5c0	0.52	0.04	2.9E-04	0	3.7E-04	0	0	0	Bacteria	Proteobacteria	Gammaproteobacteria	Xanthomonadales	Xanthomonadaceae	*Lysobacter*	
	888c1f97b0dae8c8b3264f21fa23818e	0.63	0.01	4.4E-04	1.4E-03	4.1E-03	2.0E-03	0	0	Bacteria	Verrucomicrobia	Spartobacteria	Chthoniobacterales	Chthoniobacteraceae	*DA101*	
	25976640bfc999b49f17ca08410d77ac	0.66	0.015	2.4E-03	1.5E-03	3.6E-03	7.8E-04	0	0	Bacteria	Verrucomicrobia	Spartobacteria	Chthoniobacterales	Chthoniobacteraceae	*DA101*	
	ee9c5a8f692f7c2b42c869905be38c2e	0.58	0.02	0	1.4E-03	4.9E-03	0	0	0	Bacteria	Verrucomicrobia	Spartobacteria	Chthoniobacterales	Chthoniobacteraceae	*DA101*	
	3bde92b6a319f4c6440e2cedbda93254	0.52	0.05	4.4E-04	0	7.1E-04	0	0	0	Bacteria	Verrucomicrobia	Spartobacteria	Chthoniobacterales	Chthoniobacteraceae	*DA101*	
	8a8a97e6b7d0f7046388c72d9946e332	0.54	0.05	0	2.4E-03	6.4E-03	2.5E-03	0	0	Bacteria	Verrucomicrobia	Spartobacteria	Chthoniobacterales	Chthoniobacteraceae	*DA101*	
	55b35e0d44cc327b6e23a12858516dd6	0.52	0.045	4.9E-05	1.3E-03	9.8E-05	0	0	0	Unassigned						
	119d712dd230b4ae4afb11c79714e04d	0.45	0.05	0	5.2E-04	2.7E-04	0	0	0	Unassigned						
Quercus	f2a80ca7a1f03c84fef11bfbd8c77604	0.54	0.05	0	0	0	0	4.2E-04	7.8E-05	Bacteria	Acidobacteria	Chloracidobacteria	RB41	Ellin6075		
	9f6d0ba5232b535de37fc3bf70d85d83	0.61	0.005	0	0	1.5E-04	2.2E-03	5.6E-04	2.0E-04	Bacteria	Acidobacteria	Chloracidobacteria	RB41			
	dc0a4a03916692f64e4d2cdbb730ccb5	0.69	0.005	0	0	0	1.2E-03	1.1E-03	5.1E-04	Bacteria	Acidobacteria	Chloracidobacteria	RB41			
	55695752bd3ba9bc1e262b6737f64b97	0.54	0.03	0	0	0	0	8.1E-04	5.5E-04	Bacteria	Acidobacteria	Acidobacteria-6	iii1-15			
	6d719ad12060a828dcf712d2a41b612e	0.60	0.04	2.4E-04	0	7.6E-04	2.9E-04	2.6E-03	7.0E-04	Bacteria	Acidobacteria	Acidobacteria-6	iii1-15			
	a54ccadffd4fb894c10fcffacc096aa5	0.54	0.05	0	0	0	0	8.1E-04	5.9E-04	Bacteria	Acidobacteria	Acidobacteria-6	iii1-15			
	dc4919160d9fc1a6cc69b05439c4ccbc	0.56	0.05	0	0	2.2E-04	5.9E-04	6.4E-04	1.7E-03	Bacteria	Acidobacteria	Acidobacteria-6	iii1-15			
	b9fcec01f1c5272ff219ee681eb39d58	0.57	0.05	0	0	3.4E-04	2.5E-03	2.6E-03	6.3E-04	Bacteria	Acidobacteria	Acidobacteriia	Acidobacteriales	Acidobacteriaceae	*Granulicella*	*paludicola*
	8ee6c4ab30ebefae1d1861564e7ff7a8	0.59	0.02	0	0	0	0	1.7E-03	8.2E-04	Bacteria	Acidobacteria	Solibacteres	Solibacterales	Solibacteraceae	*Candidatus Solibacter*	
	29336d1aff2b352997780a7fbc53f1b8	0.54	0.04	0	0	0	1.5E-04	2.0E-04	2.7E-04	Bacteria	Acidobacteria	Sva0725	Sva0725			
	1839e46bb8e4d01c6dc3fb75a2469389	0.54	0.035	0	0	0	4.9E-05	1.5E-04	6.3E-04	Bacteria	Actinobacteria	Acidimicrobiia	Acidimicrobiales	C111		
	7353297d3e40e11fcaf6bb99a80902a3	0.54	0.05	0	0	0	7.8E-04	5.6E-04	1.2E-04	Bacteria	Actinobacteria	Acidimicrobiia	Acidimicrobiales	EB1017		
	b37d61f2081038a5c8e89db3bc36d348	0.64	0.01	0	0	0	0	9.8E-05	7.8E-04	Bacteria	Actinobacteria	Acidimicrobiia	Acidimicrobiales			
	ad574a0549476e3fe09be14e6cbee475	0.77	0.015	3.9E-04	3.9E-04	1.2E-04	4.4E-04	7.6E-04	2.8E-03	Bacteria	Actinobacteria	Actinobacteria	Actinomycetales	Cellulomonadaceae	*Cellulomonas*	
	c95ae406e809bf890d9c57ac747a1904	0.54	0.025	0	0	0	0	2.2E-04	7.8E-04	Bacteria	Actinobacteria	Actinobacteria	Actinomycetales	Intrasporangiaceae	*Phycicoccus*	
	d52c105b9586ddf8b1adf5800d7820db	0.58	0.05	4.4E-04	0	0	6.4E-04	6.1E-04	3.1E-04	Bacteria	Actinobacteria	Actinobacteria	Actinomycetales	Kineosporiaceae		
	7a52b9e5295f9aa5d98040cea1b5dc4d	0.59	0.03	0	0	0	0	4.4E-04	2.5E-03	Bacteria	Actinobacteria	Actinobacteria	Actinomycetales	Microbacteriaceae		
	cbd3bd0c85ddab9285651fccd2878632	0.64	0.01	0	0	0	0	1.1E-03	2.5E-03	Bacteria	Actinobacteria	Actinobacteria	Actinomycetales	Nakamurellaceae		
	1693b72fb96d3fa45f67065ef3445042	0.64	0.015	0	0	0	0	5.4E-04	1.9E-03	Bacteria	Actinobacteria	Actinobacteria	Actinomycetales	Nakamurellaceae		
	b40e445e1ad7edc719d9c2c850533e7c	0.54	0.05	0	0	0	0	4.9E-05	8.6E-04	Bacteria	Actinobacteria	Actinobacteria	Actinomycetales	Nocardioidaceae	*Nocardioides*	
	5a78a8936d287d363796fcce0c138d48	0.64	0.005	0	0	0	0	5.9E-04	1.4E-03	Bacteria	Actinobacteria	Actinobacteria	Actinomycetales	Nocardioidaceae		
	b5c5886ff266ea4b0929cabe7b6e8329	0.59	0.02	0	0	0	0	1.6E-03	1.6E-03	Bacteria	Actinobacteria	Actinobacteria	Actinomycetales	Nocardioidaceae		
	f7bb60140fc3a1fb648c0f18b8c6d36c	0.59	0.02	0	0	0	0	5.6E-04	9.4E-04	Bacteria	Actinobacteria	Actinobacteria	Actinomycetales	Pseudonocardiaceae	*Actinomycetospora*	
	49a1897b3e8a961a219716df9d1892f5	0.62	0.03	5.9E-04	0	0	0	3.3E-03	7.8E-04	Bacteria	Actinobacteria	Actinobacteria	Actinomycetales	Pseudonocardiaceae	*Actinomycetospora*	
	4d66ee483deffc2e46ce3c5a29abf062	0.54	0.04	0	0	0	0	1.5E-04	3.5E-04	Bacteria	Actinobacteria	Actinobacteria	Actinomycetales			
	42ba659e0465b5fa83a90c4c10c787c6	0.54	0.04	0	0	0	0	1.5E-04	3.9E-04	Bacteria	Actinobacteria	MB-A2-108	0319-7L14			
	0222d3a73fafa53c994878a023dc1efc	0.59	0.015	0	0	0	0	4.6E-04	6.3E-04	Bacteria	Actinobacteria	Thermoleophilia	Gaiellales	Gaiellaceae		
	748f5e1091680b0ee4428fc49c6ad325	0.61	0.045	1.5E-04	0	9.8E-05	0	6.4E-04	1.5E-03	Bacteria	Actinobacteria	Thermoleophilia	Gaiellales	Gaiellaceae		
	b3997a39a26f08d316eb5db73755b147	0.59	0.015	0	0	0	0	5.4E-04	3.1E-04	Bacteria	Actinobacteria	Thermoleophilia	Solirubrobacterales			
	7591f84c80f89d88ecfe4b06dd7316aa	0.59	0.02	0	0	0	1.1E-03	8.3E-04	5.1E-04	Bacteria	Bacteroidetes	Saprospirae	Saprospirales	Chitinophagaceae	*Niabella*	
	d53e59e92eb37b67e7bd24d82ce5b642	0.69	0.005	0	0	0	1.5E-04	6.4E-04	9.8E-04	Bacteria	Bacteroidetes	Saprospirae	Saprospirales	Chitinophagaceae		
	0fa8f80e7ba892e2ec52157157d86c55	0.64	0.015	0	0	0	0	4.9E-04	2.2E-03	Bacteria	Bacteroidetes	Saprospirae	Saprospirales	Chitinophagaceae		
	5c7efb16bc98732254198caeb4f7b6f1	0.59	0.02	0	0	0	0	4.6E-04	5.5E-04	Bacteria	Bacteroidetes	Saprospirae	Saprospirales	Chitinophagaceae		
	1a7a59e2f294f382b06f31afe0f07a54	0.62	0.025	1.5E-04	0	0	1.5E-03	2.2E-04	5.1E-04	Bacteria	Bacteroidetes	Saprospirae	Saprospirales	Chitinophagaceae		
	f6baa31e561aa1e73cebe053ad8121cb	0.54	0.03	0	0	0	2.9E-04	1.1E-03	0	Bacteria	Bacteroidetes	Saprospirae	Saprospirales	Chitinophagaceae		
	8b2e4b0d8be925fbdd72f1143f712d16	0.54	0.04	0	0	0	0	1.7E-04	1.9E-03	Bacteria	Bacteroidetes	Saprospirae	Saprospirales	Chitinophagaceae		
	291713113d06d0a68de25a552b310a23	0.54	0.045	0	0	0	0	5.9E-04	1.1E-03	Bacteria	Bacteroidetes	Saprospirae	Saprospirales	Chitinophagaceae		
	3676f2751212b17db73ceb4c71ac2264	0.60	0.05	0	0	3.2E-04	9.8E-05	6.1E-04	8.2E-04	Bacteria	Bacteroidetes	Saprospirae	Saprospirales	Chitinophagaceae		
	3b96945028ffab801fb5d7a8caf5f257	0.54	0.05	0	0	0	2.4E-04	4.9E-05	7.8E-05	Bacteria	Bacteroidetes	Saprospirae	Saprospirales	Chitinophagaceae		
	a41d967e97b5a6e331173bab846605d9	0.56	0.05	0	1.6E-03	0	2.9E-03	5.6E-04	3.9E-04	Bacteria	Bacteroidetes	Saprospirae	Saprospirales	Chitinophagaceae		
	5e301f77b78181286f42e00aed5c6719	0.61	0.035	0	0	1.2E-04	4.9E-05	9.3E-04	4.3E-04	Bacteria	Bacteroidetes	Saprospirae	Saprospirales	Saprospiraceae		
	0a4e4c32fe6701fb6bba23b296562579	0.59	0.035	0	0	0	2.0E-04	5.6E-04	1.1E-03	Bacteria	Bacteroidetes	Cytophagia	Cytophagales	Cytophagaceae	*Adhaeribacter*	
	24aa511e07dcd75479f64bbf0f76b5f4	0.54	0.05	0	0	0	0	1.1E-03	3.5E-04	Bacteria	Bacteroidetes	Cytophagia	Cytophagales	Cytophagaceae	*Adhaeribacter*	
	23b870d0574883d71d3be8efbb227f91	0.63	0.015	9.8E-05	0	0	0	3.2E-04	1.7E-03	Bacteria	Bacteroidetes	Cytophagia	Cytophagales	Cytophagaceae		
	8aa440a88e8c2668f734d21f78ee17ca	0.54	0.03	0	0	0	2.0E-04	1.1E-03	0	Bacteria	Bacteroidetes	Flavobacteriia	Flavobacteriales	Flavobacteriaceae	*Flavobacterium*	
	4caf855972a2f2edefc5138c4f8d0ecc	0.57	0.045	3.9E-04	0	0	0	2.1E-03	4.7E-04	Bacteria	Bacteroidetes	Sphingobacteriia	Sphingobacteriales	Sphingobacteriaceae		
	0dfb5cdacfcc457d315037568f5da372	0.59	0.02	0	0	0	0	2.2E-04	2.0E-04	Bacteria	Bacteroidetes	Sphingobacteriia	Sphingobacteriales			
	da150aff3d59124c280b5fd8472c550e	0.73	0.005	0	0	0	0	5.3E-03	2.7E-03	Bacteria	Chlamydiae	Chlamydiia	Chlamydiales	Rhabdochlamydiaceae	*Candidatus Rhabdochlamydia*	
	e77e7ca09de4e691738c41f6d22c6fa7	0.78	0.005	2.0E-04	0	1.2E-04	1.5E-03	1.4E-03	2.5E-03	Bacteria	Chloroflexi	Ellin6529				
	8b0ba802609422879ab78f9b6dd5fd7e	0.54	0.05	0	0	0	2.9E-04	1.7E-04	7.8E-05	Bacteria	Gemmatimonadetes	Gemm-5				
	4eee045d602a14ff24aef24d80ec2eb5	0.59	0.025	0	0	0	0	6.4E-04	2.7E-04	Bacteria	Gemmatimonadetes	Gemmatimonadetes	Ellin5290			
	a8e8ce2197d3947a0769abfc507cf46c	0.58	0.04	0	0	2.4E-05	0	2.9E-04	3.9E-04	Bacteria	Planctomycetes	Planctomycetia	Gemmatales	Gemmataceae	*Gemmata*	
	73cd0b59d3c1ee6c6470bd953cc007a2	0.58	0.05	0	0	4.9E-05	1.3E-03	1.7E-04	2.3E-04	Bacteria	Planctomycetes	Planctomycetia	Gemmatales	Gemmataceae		
	0126dc4bf3e31b554a55aaeefb3deb18	0.59	0.03	0	0	0	0	3.9E-04	8.6E-04	Bacteria	Planctomycetes	Planctomycetia	Planctomycetales	Planctomycetaceae	*Planctomyces*	
	114159617d8501c001b0fee1fac85cf3	0.84	0.005	0	0	0	5.2E-03	9.8E-04	7.4E-04	Bacteria	Proteobacteria	Alphaproteobacteria	Rhizobiales	Hyphomicrobiaceae	*Rhodoplanes*	
	6598429447ed4906911ed1b36f9c17ca	0.59	0.02	0	0	0	0	2.3E-03	1.4E-03	Bacteria	Proteobacteria	Alphaproteobacteria	Rhizobiales	Hyphomicrobiaceae	*Rhodoplanes*	
	d46be10411d895030f89392b6f352e5f	0.54	0.04	0	0	0	4.9E-04	6.8E-04	0	Bacteria	Proteobacteria	Alphaproteobacteria	Rhizobiales	Hyphomicrobiaceae	*Rhodoplanes*	
	a9a07cbb160266ff09cf49ad5d8b8c9a	0.54	0.045	0	0	0	0	4.9E-04	4.3E-04	Bacteria	Proteobacteria	Alphaproteobacteria	Rhizobiales	Hyphomicrobiaceae	*Rhodoplanes*	
	14331f18e634d3684c9185733a8c7be7	0.68	0.01	1.5E-04	0	0	5.8E-03	8.1E-04	6.3E-04	Bacteria	Proteobacteria	Alphaproteobacteria	Rhizobiales	Methylocystaceae		
	16357c104069b5c62302e913480dce46	0.54	0.03	0	0	0	0	2.4E-04	6.3E-04	Bacteria	Proteobacteria	Alphaproteobacteria	Rhodobacterales	Rhodobacteraceae	*Rhodobacter*	
	34f1a1134a2f5433fd6b90de56403842	0.73	0.005	0	7.8E-04	0	2.1E-03	1.1E-03	1.8E-03	Bacteria	Proteobacteria	Alphaproteobacteria	Rhodospirillales	Rhodospirillaceae	*Reyranella*	*massiliensis*
	ffb5ee2acc41ed03375dfbebe75581a0	0.54	0.04	0	0	0	6.8E-04	9.8E-05	1.6E-03	Bacteria	Proteobacteria	Alphaproteobacteria	Sphingomonadales	Erythrobacteraceae		
	947bb131c525264f69000efd5ad1fd83	0.64	0.015	0	0	0	1.5E-04	1.2E-03	2.3E-04	Bacteria	Proteobacteria	Alphaproteobacteria	Sphingomonadales	Sphingomonadaceae	*Kaistobacter*	
	939c0f06b9e292aee1001d3e1e9f399c	0.59	0.02	0	0	0	1.1E-03	9.5E-04	6.6E-04	Bacteria	Proteobacteria	Alphaproteobacteria	Sphingomonadales	Sphingomonadaceae	*Kaistobacter*	
	d0811aea45b2734c3fae658d086f6815	0.60	0.05	7.8E-04	0	0	0	2.9E-03	7.4E-04	Bacteria	Proteobacteria	Alphaproteobacteria	Sphingomonadales	Sphingomonadaceae	*Sphingomonas*	*wittichii*
	0f86116fc894194f6b9fc85912b8c214	0.59	0.02	0	0	0	0	5.9E-04	4.3E-04	Bacteria	Proteobacteria	Betaproteobacteria	A21b	UD5		
	d9d980e45e7118c91301ed3c168b9af7	0.69	0.005	0	0	0	0	3.1E-03	4.9E-03	Bacteria	Proteobacteria	Betaproteobacteria	Burkholderiales	Comamonadaceae		
	3021727b4ea286a95c064fe15d2a7045	0.54	0.05	0	0	0	0	9.0E-04	3.1E-04	Bacteria	Proteobacteria	Betaproteobacteria	Ellin6067			
	913e48d9e2caae8fdb73d2822a52a26b	0.54	0.025	0	0	0	1.5E-04	2.2E-04	7.8E-04	Bacteria	Proteobacteria	Betaproteobacteria	MND1			
	b5eaefe73e0af247dcdaeea2f5ce433b	0.54	0.04	0	0	0	1.2E-03	5.6E-04	0	Bacteria	Proteobacteria	Betaproteobacteria	MND1			
	92c4c226a21b38307613de2fc95f55b5	0.75	0.005	0	2.6E-04	0	2.2E-03	8.6E-04	5.9E-04	Bacteria	Proteobacteria	Betaproteobacteria	SC-I-84			
	ad4db7a139d3fd19f38897fa9dee062f	0.63	0.015	2.0E-04	0	0	1.5E-03	1.9E-03	0	Bacteria	Proteobacteria	Betaproteobacteria	SC-I-84			
	e348dd0f8b01220a2d4fc152bc8feb4e	0.64	0.015	0	0	0	0	1.4E-03	7.4E-04	Bacteria	Proteobacteria	Betaproteobacteria	SC-I-84			
	7d590b2caa2ebf22f55cc9b5e1ddc996	0.59	0.03	0	0	2.4E-04	6.8E-04	5.6E-04	8.2E-04	Bacteria	Proteobacteria	Betaproteobacteria	SC-I-84			
	2ef6e4e62813fdf7c2f8edc5bfc59a1f	0.69	0.005	0	0	0	6.8E-04	7.8E-04	1.2E-04	Bacteria	Proteobacteria	Betaproteobacteria				
	68dcb051543b40c92375b62558e18e2a	0.64	0.015	0	0	0	6.4E-04	1.1E-03	5.1E-04	Bacteria	Proteobacteria	Betaproteobacteria				
	0e14720959023e974580467871fee963	0.54	0.04	0	0	0	9.8E-05	2.4E-05	1.0E-03	Bacteria	Proteobacteria	Deltaproteobacteria	Desulfuromonadales	Geobacteraceae	*Geobacter*	
	fe5d52e09d17524c0df9d38edc91024d	0.54	0.05	0	0	0	1.5E-04	1.7E-04	3.5E-04	Bacteria	Proteobacteria	Deltaproteobacteria	Myxococcales	Cystobacterineae		
	903054790ae2d546c6f832a1765b4c93	0.63	0.025	5.9E-04	1.2E-03	0	2.4E-03	3.0E-03	1.2E-03	Bacteria	Proteobacteria	Deltaproteobacteria	Myxococcales	Haliangiaceae		
	265f6c0f5ed7eb2a0ac39225f15e3968	0.58	0.03	0	0	4.9E-05	7.8E-04	3.2E-04	2.7E-04	Bacteria	Proteobacteria	Deltaproteobacteria	Myxococcales	Polyangiaceae		
	cd438737c067248349550723030bec5b	0.67	0.005	0	0	7.3E-05	5.9E-04	1.4E-03	3.9E-04	Bacteria	Proteobacteria	Deltaproteobacteria	Myxococcales			
	1099baeea6ed291cebfdd3b909a0fb9b	0.59	0.02	0	0	0	8.8E-04	6.6E-04	9.0E-04	Bacteria	Proteobacteria	Deltaproteobacteria	Myxococcales			
	48da65a617b0a0a0a4d87ce9b9ce1ab6	0.59	0.02	0	0	0	0	4.6E-04	5.5E-04	Bacteria	Proteobacteria	Deltaproteobacteria	Myxococcales			
	5c6f0b2e364269f1f4bf502ad1af3706	0.53	0.04	4.9E-05	0	0	6.8E-04	4.9E-04	1.6E-04	Bacteria	Proteobacteria	Deltaproteobacteria	Myxococcales			
	ca7edd2c0c85a7b9898a8e641b457e50	0.54	0.045	0	0	0	0	1.7E-03	3.5E-04	Bacteria	Proteobacteria	Deltaproteobacteria	Myxococcales			
	d80072309ff587c9619b9be29537c8fe	0.54	0.035	0	0	0	0	5.6E-04	7.8E-04	Bacteria	Proteobacteria	Gammaproteobacteria	Thiotrichales	Piscirickettsiaceae		
	ef86c96c0c0e8ac38381b5ca65b1a1d6	0.59	0.02	0	0	0	4.9E-04	9.3E-04	3.9E-05	Bacteria	Proteobacteria	Gammaproteobacteria	Xanthomonadales	Sinobacteraceae		
	8460d749c54a4dd2cde6dd7c7388d686	0.54	0.05	0	0	0	0	8.3E-04	1.6E-04	Bacteria	Proteobacteria	Gammaproteobacteria	Xanthomonadales	Sinobacteraceae		
	03af51e75c7ee46771685036b7c12f28	0.59	0.01	0	0	0	3.4E-04	6.4E-04	7.8E-04	Bacteria	Proteobacteria	Gammaproteobacteria	Xanthomonadales	Xanthomonadaceae		
	32f853714a852ee9fe3f333224dcbf72	0.59	0.02	0	0	0	9.8E-05	4.9E-04	1.6E-04	Bacteria	Proteobacteria	Gammaproteobacteria	Xanthomonadales	Xanthomonadaceae		
	57f3562d588b974e7979619da315d1f6	0.64	0.01	0	0	0	5.9E-04	4.9E-04	2.0E-04	Bacteria	Verrucomicrobia	Spartobacteria	Chthoniobacterales	Chthoniobacteraceae	*Ellin506*	
	9bcdfc8204acdd298aba85b062603595	0.61	0.04	0	0	7.3E-05	0	4.6E-04	7.0E-04	Bacteria	Verrucomicrobia	Spartobacteria	Chthoniobacterales	Chthoniobacteraceae	*Ellin506*	

	22d0ce457b2606ee94ce5ead360574bc	0.61	0.035	0	0	2.2E-04	0	1.3E-03	1.5E-03	Bacteria	Verrucomicrobia	Verrucomicrobiae	Verrucomicrobiales	Verrucomicrobiaceae	*Luteolibacter*	
	fba4613944482bd49c4f9cbe2f6c02a4	0.59	0.02	0	0	0	0	3.2E-04	2.3E-04	Bacteria	WS3	PRR-12	Sediment-1			
