# Supplementary material for: Invasive vegetation affects amphibian skin microbiota and body condition
Source: PeerJ. 2020 Feb 19;8:e8549. doi: 10.7717/peerj.8549 (PMC7035873; doi:10.7717/peerj.8549)
Supplement: Supplemental Information 1 [file peerj-08-8549-s001.zip › PeerJ Supp/TableS2.pdf]

Assignment	ASV ID	LDA stats		*Eucalyptus* Sites			*Quercus* sites			Taxonomy						
		LDA	p.value	E1	E2	E3	N1	N2	N3	Kingdom	Phylum	Class	Order	Family	Genus	Species
Eucalyptus	df0534866a33108ad427b73b709ebfe8	0.63	0.005	1.2E-03	3.6E-04	5.5E-04	9.8E-05	7.8E-05	0	Bacteria	Acidobacteria	Acidobacteriia	Acidobacteriales	Acidobacteriaceae		
	e2d9476d14203066aa5c966011252216	0.62	0.015	9.8E-04	5.6E-05	1.0E-03	0	2.0E-05	0	Bacteria	Acidobacteria	Acidobacteriia	Acidobacteriales	Acidobacteriaceae		
	b379b1521d1a107507e12b3c376067b0	0.56	0.03	0	3.6E-04	1.0E-03	0	3.9E-05	0	Bacteria	Acidobacteria	iii1-8	DS-18			
	15d0c75db0d4705fd5dd4770a59c8f00	0.65	0.005	2.0E-04	7.0E-04	1.2E-03	0	0	0	Bacteria	Actinobacteria	Acidimicrobiia	Acidimicrobiales	EB1017		
	5f5dc2afebad265a4e4db64483f3f704	0.50	0.025	9.8E-05	1.4E-04	4.6E-04	0	0	0	Bacteria	Actinobacteria	Acidimicrobiia	Acidimicrobiales	Microthrixaceae		
	fcdee861c3a5b2efd76eb7d3ab1d606c	0.45	0.05	2.0E-04	5.9E-04	7.1E-05	0	3.9E-05	0	Bacteria	Actinobacteria	Actinobacteria	Actinomycetales	Microbacteriaceae	*Microbacterium*	
	20e7ada7880473eadf42573519350147	0.57	0.005	3.2E-03	6.8E-03	5.0E-04	0	0	0	Bacteria	Actinobacteria	Actinobacteria	Actinomycetales	Micrococcaceae		
	477cc94cc17f6999ea63444cacaa470a	0.45	0.05	7.4E-04	4.2E-04	2.3E-04	0	7.8E-05	0	Bacteria	Actinobacteria	Actinobacteria	Actinomycetales	Nocardioidaceae	*Nocardioides*	
	3803258f892a9f8b6ee2e1b6de8436d4	0.53	0.025	1.1E-03	4.5E-04	2.5E-04	0	0	0	Bacteria	Actinobacteria	Actinobacteria	Actinomycetales	Nocardioidaceae		
	7faccb65676a2c40636da004a18ec5a0	0.50	0.05	0	2.8E-04	1.1E-03	0	1.6E-04	0	Bacteria	Actinobacteria	Actinobacteria	Actinomycetales	Nocardioidaceae		
	113b318d23356092ddae70f544df4718	0.46	0.035	7.8E-05	2.2E-04	3.6E-04	0	0	0	Bacteria	Actinobacteria	Actinobacteria	Actinomycetales	Pseudonocardiaceae	*Pseudonocardia*	
	6ced708f78dca8c0dd166df25149feb6	0.46	0.035	5.9E-05	2.5E-04	5.7E-04	0	0	0	Bacteria	Actinobacteria	Thermoleophilia	Solirubrobacterales	Patulibacteraceae		
	4479830df8a883aa2997024fadc88b52	0.46	0.045	5.9E-04	0	8.2E-04	0	0	0	Bacteria	Bacteroidetes	Saprospirae	Saprospirales	Chitinophagaceae		
	e007858c3abd7a15cf8e8930820a689e	0.53	0.015	2.9E-04	6.1E-04	2.8E-04	0	0	0	Bacteria	Bacteroidetes	Flavobacteriia	Flavobacteriales	Flavobacteriaceae	*Epilithonimonas*	
	5984c840c5cdeabb6540d4863e81270a	0.58	0.05	2.1E-03	8.9E-04	6.8E-04	4.9E-05	4.7E-04	0	Bacteria	Bacteroidetes	Sphingobacteriia	Sphingobacteriales	Sphingobacteriaceae	*Pedobacter*	
	ad5d17028975c96a543bc1bdea4692f4	0.76	0.005	8.3E-02	2.1E-02	2.2E-02	0	1.2E-04	9.8E-05	Bacteria	Chlamydiae	Chlamydiia	Chlamydiales	Chlamydiaceae		
	411512cc5d5ff7a557daeafffbfcc0dd	0.42	0.045	0.0E+00	5.6E-05	1.1E-04	0	0	0	Bacteria	Planctomycetes	Planctomycetia	Pirellulales	Pirellulaceae		
	371675b527b024b935987d984e5e0adf	0.50	0.02	1.2E-04	1.4E-04	6.6E-04	0	0	0	Bacteria	Proteobacteria	Alphaproteobacteria	Rhizobiales	Hyphomicrobiaceae	*Hyphomicrobium*	
	33fd454253b9038b04dc90dc9902d0dc	0.46	0.035	2.3E-04	3.4E-04	4.3E-04	0	0	0	Bacteria	Proteobacteria	Alphaproteobacteria	Rhizobiales	Hyphomicrobiaceae	*Pedomicrobium*	
	00e5c53e5de89d9e75ef2771ed62717f	0.55	0.015	8.0E-04	3.9E-04	2.8E-03	0	6.3E-04	0	Bacteria	Proteobacteria	Alphaproteobacteria	Rhizobiales	Hyphomicrobiaceae	*Rhodoplanes*	
	13d713ae52a5bf5fddfa3b92add63266	0.46	0.03	1.2E-04	1.4E-04	1.4E-04	0	0	0	Bacteria	Proteobacteria	Alphaproteobacteria	Rhizobiales	Hyphomicrobiaceae		
	8f496bedd06ec30e808cda1970316225	0.42	0.045	1.6E-04	1.1E-04	2.1E-04	0	0	0	Bacteria	Proteobacteria	Alphaproteobacteria	Rhizobiales	Methylobacteriaceae	*Methylobacterium*	*adhaesivum*
	a552c538964597136b53d5f7881f41e1	0.60	0.01	8.2E-04	1.1E-04	1.5E-03	0	0	0	Bacteria	Proteobacteria	Alphaproteobacteria	Rhizobiales	Methylocystaceae		
	14331f18e634d3684c9185733a8c7be7	0.68	0.025	2.7E-04	2.0E-03	2.4E-03	2.0E-03	2.3E-04	7.3E-05	Bacteria	Proteobacteria	Alphaproteobacteria	Rhizobiales	Methylocystaceae		
	980978a371cc5942be087bf324c5e2f3	0.57	0.005	7.2E-04	1.7E-04	3.0E-04	0	0	0	Bacteria	Proteobacteria	Alphaproteobacteria	Rhodospirillales	Acetobacteraceae	*Acidocella*	
	822754e7f9add2d17f33255c21748e29	0.53	0.025	0	2.5E-04	2.0E-04	0	0	4.9E-05	Bacteria	Proteobacteria	Alphaproteobacteria	Rhodospirillales	Rhodospirillaceae		
	365ebfdf1a370ffbd233bf0dc8e22cab	0.63	0.01	2.9E-03	9.8E-04	5.7E-04	0	5.9E-05	3.2E-04	Bacteria	Proteobacteria	Alphaproteobacteria	Sphingomonadales	Sphingomonadaceae	*Blastomonas*	
	26dced51f936f6ac6f6200bd49079161	0.71	0.005	6.5E-04	1.5E-03	6.3E-03	0	0	0	Bacteria	Proteobacteria	Alphaproteobacteria	Sphingomonadales	Sphingomonadaceae	*Novosphingobium*	
	42630c98d41bbf95b2a504084602f221	0.46	0.045	7.0E-04	1.4E-04	0	0	0	0	Bacteria	Proteobacteria	Alphaproteobacteria	Sphingomonadales	Sphingomonadaceae	*Sphingomonas*	*wittichii*
	2899612d084dff709dc7a2973a7121f6	0.46	0.03	4.7E-04	1.4E-04	1.4E-04	0	0	0	Bacteria	Proteobacteria	Betaproteobacteria	Burkholderiales			
	999e7806b23a27b7759c33ac170411f7	0.64	0.005	3.9E-04	9.2E-04	1.7E-03	0	1.4E-04	0	Bacteria	Proteobacteria	Betaproteobacteria	IS-44			
	2511ff5d78ef6d53e9819a09ca5d9eea	0.53	0.01	5.9E-04	2.0E-04	3.9E-04	0	0	0	Bacteria	Proteobacteria	Deltaproteobacteria	Myxococcales			
	a3b2563c2e0b9dc498975725b75fc560	0.50	0.035	2.3E-04	1.1E-03	5.9E-04	0	0	0	Bacteria	Proteobacteria	Gammaproteobacteria	Xanthomonadales	Sinobacteraceae	*Steroidobacter*	
	c164fd002b5502c5963286a9322833a0	0.45	0.05	2.9E-04	9.8E-04	8.4E-04	0	0	1.5E-04	Bacteria	Proteobacteria	Gammaproteobacteria	Xanthomonadales	Sinobacteraceae	*Steroidobacter*	
	8ce5d403015616c859b6c39e84bc84ff	0.50	0.045	5.9E-05	3.6E-04	8.5E-04	3.4E-04	0	0	Bacteria	Proteobacteria	Gammaproteobacteria	Xanthomonadales	Sinobacteraceae		
	81a2e8f0dcd6a54d98fc92de96dd44a2	0.57	0.005	8.2E-04	3.9E-04	1.8E-04	0	0	0	Unassigned						
	82b1125503380c29941c63bbe7482559	0.48	0.015	0	0	0	1.3E-03	1.4E-04	0	Archaea	Crenarchaeota	Thaumarchaeota	Nitrososphaerales	Nitrososphaeraceae	*Candidatus Nitrososphaera*	*SCA1170*
	dc0a4a03916692f64e4d2cdbb730ccb5	0.48	0.015	0	0	0	2.0E-04	2.7E-04	4.9E-05	Bacteria	Acidobacteria	Chloracidobacteria	RB41			
	9f6d0ba5232b535de37fc3bf70d85d83	0.51	0.02	0	0	1.8E-05	9.8E-05	1.4E-04	2.9E-04	Bacteria	Acidobacteria	Chloracidobacteria	RB41			
	25368dd1216da1cd95e52e114599cec0	0.64	0.005	0	0	0	2.4E-04	1.2E-04	9.8E-04	Bacteria	Acidobacteria	Acidobacteria-5				
	787f0b4eca2776727f75985f671b52bc	0.48	0.015	0	0	0	2.4E-04	7.8E-05	2.7E-04	Bacteria	Acidobacteria	Acidobacteria-6	CCU21			
	621d1089ed7a8550ac6fe480340f6951	0.66	0.01	9.8E-05	1.1E-04	3.7E-04	0	1.7E-03	1.4E-03	Bacteria	Acidobacteria	Acidobacteria-6	iii1-15	mb2424		
	11eda7d42819e5bcde46237d083872da	0.48	0.005	0	0	0	1.5E-03	0.0E+00	1.5E-03	Bacteria	Acidobacteria	Acidobacteria-6	iii1-15			
	55695752bd3ba9bc1e262b6737f64b97	0.48	0.01	0	0	0	5.4E-04	5.3E-04	2.9E-04	Bacteria	Acidobacteria	Acidobacteria-6	iii1-15			
	af887236ee0501cd8d66abac069301cd	0.52	0.01	0	0	0	3.4E-04	3.9E-05	6.1E-04	Bacteria	Acidobacteria	Acidobacteria-6	iii1-15			
	dfa6be6ab09b40cf408366b32d58f694	0.62	0.01	1.4E-04	0	3.4E-04	6.4E-04	2.0E-04	2.1E-03	Bacteria	Acidobacteria	Acidobacteria-6	iii1-15			
	1f6720450a1dc9d37c5dadc071c4c07f	0.57	0.02	0	0	3.6E-04	0	6.3E-04	6.6E-04	Bacteria	Acidobacteria	Acidobacteria-6	iii1-15			
	a54ccadffd4fb894c10fcffacc096aa5	0.43	0.04	0	0	0	0	3.9E-04	0	Bacteria	Acidobacteria	Acidobacteria-6	iii1-15			
	3298a670bce92abe1ed8da51b23323cb	0.47	0.035	0	0	1.8E-05	6.8E-04	5.9E-05	1.2E-04	Bacteria	Acidobacteria	Acidobacteriia	Acidobacteriales	Acidobacteriaceae	*Granulicella*	*tundricola*
	d60cbce30d332606d32e57400d749dfd	0.62	0.015	5.9E-05	2.2E-04	1.2E-04	2.0E-04	3.1E-04	1.4E-03	Bacteria	Acidobacteria	Solibacteres	Solibacterales	Bryobacteraceae		
	1349d393b150b4cea3ad09dc54c76810	0.49	0.045	0	0	1.1E-04	7.8E-04	0	5.6E-04	Bacteria	Acidobacteria	Solibacteres	Solibacterales	Solibacteraceae	*Candidatus Solibacter*	
	1839e46bb8e4d01c6dc3fb75a2469389	0.63	0.005	0	0	3.4E-04	3.9E-04	3.3E-04	8.6E-04	Bacteria	Actinobacteria	Acidimicrobiia	Acidimicrobiales	C111		
	7b1dc4f4c74f9ce4c60cefad4d362fc9	0.67	0.005	0	0	0	2.0E-04	4.3E-04	1.3E-03	Bacteria	Actinobacteria	Acidimicrobiia	Acidimicrobiales	C111		
	28c2c8fd4647cdb4d35caae32d2a7f21	0.47	0.01	0	0	1.8E-05	2.0E-04	3.5E-04	7.3E-05	Bacteria	Actinobacteria	Acidimicrobiia	Acidimicrobiales	C111		
	1bbcef41d756f879c072c9de9035841e	0.53	0.015	2.0E-05	0	5.3E-05	0	3.1E-04	2.9E-04	Bacteria	Actinobacteria	Acidimicrobiia	Acidimicrobiales	EB1017		
	bba6a064d4862a5ba10b68bbb8ce4014	0.43	0.035	0	0	0	0	2.3E-04	2.2E-04	Bacteria	Actinobacteria	Acidimicrobiia	Acidimicrobiales	EB1017		
	0ecdac55b8e71511c514118efa24f42f	0.37	0.05	0	0	0	4.4E-04	0	1.5E-04	Bacteria	Actinobacteria	Acidimicrobiia	Acidimicrobiales	EB1017		
	65f23a9351c81cf10a25003861eb9830	0.74	0.005	7.8E-05	8.4E-05	3.6E-05	3.4E-04	6.5E-04	1.3E-03	Bacteria	Actinobacteria	Acidimicrobiia	Acidimicrobiales	Iamiaceae	*Iamia*	
	0c02825c675230626fc5d99415098426	0.74	0.005	0	2.0E-04	3.9E-04	2.0E-04	7.4E-04	1.4E-03	Bacteria	Actinobacteria	Acidimicrobiia	Acidimicrobiales			
	d739ec728a0cbb0b96265f1e83eddb5b	0.48	0.01	0	0	0	0	0.0E+00	6.1E-04	Bacteria	Actinobacteria	Acidimicrobiia	Acidimicrobiales			
	0f126ee699e34f6e76d86fbe51533cd4	0.54	0.015	5.9E-05	2.8E-05	0	0	1.6E-04	5.6E-04	Bacteria	Actinobacteria	Acidimicrobiia	Acidimicrobiales			
	1c4f968b1ab83a3d38b32241322421b7	0.51	0.005	3.9E-05	0	0	0	4.3E-04	6.4E-04	Bacteria	Actinobacteria	Actinobacteria	Acidimicrobiales	Lamiaceae		
	e7aad5c85c90e80166e4b24e6f414524	0.76	0.005	0	0	5.3E-05	2.9E-04	7.8E-05	1.7E-03	Bacteria	Actinobacteria	Actinobacteria	Actinomycetales	Cellulomonadaceae	*Demequina*	
	9f2201eb779ed2b2221a73b75e2a9a7c	0.37	0.05	0	0	0	0	3.3E-04	0	Bacteria	Actinobacteria	Actinobacteria	Actinomycetales	Cryptosporangiaceae	*Cryptosporangium*	
	c95ae406e809bf890d9c57ac747a1904	0.71	0.005	0	0	2.7E-04	3.4E-04	1.9E-03	1.2E-03	Bacteria	Actinobacteria	Actinobacteria	Actinomycetales	Intrasporangiaceae	*Phycicoccus*	
	35f9837ef2d53bdbf30d1423ab06d28c	0.48	0.03	0	0	0	0	5.3E-04	0	Bacteria	Actinobacteria	Actinobacteria	Actinomycetales	Intrasporangiaceae	*Terracoccus*	
	1a1b68c68eeba6cbecbf1b1fdc5761bf	0.50	0.035	5.9E-05	0	1.2E-04	0	1.6E-03	0	Bacteria	Actinobacteria	Actinobacteria	Actinomycetales	Intrasporangiaceae		
	d52c105b9586ddf8b1adf5800d7820db	0.53	0.03	1.8E-04	0	2.8E-04	4.9E-04	4.1E-04	8.3E-04	Bacteria	Actinobacteria	Actinobacteria	Actinomycetales	Kineosporiaceae		
	03cc90a76e65874ff04ccb1a8ee2d9b4	0.54	0.04	2.3E-04	3.9E-04	1.4E-04	0	1.6E-03	5.4E-04	Bacteria	Actinobacteria	Actinobacteria	Actinomycetales	Microbacteriaceae	*Frigoribacterium*	
	7a52b9e5295f9aa5d98040cea1b5dc4d	0.72	0.005	0	1.7E-04	2.0E-04	1.3E-03	5.5E-04	4.5E-03	Bacteria	Actinobacteria	Actinobacteria	Actinomycetales	Microbacteriaceae		
	33d5a2c7d222f0b503a88a922c1312e1	0.55	0.01	3.7E-04	0	3.2E-04	1.2E-03	2.3E-03	0	Bacteria	Actinobacteria	Actinobacteria	Actinomycetales	Microbacteriaceae		
	823544d0615ab57c8d5cd62215c4c785	0.37	0.05	0	0	0	0	0	5.1E-04	Bacteria	Actinobacteria	Actinobacteria	Actinomycetales	Micromonosporaceae		
	9cbc26f9122646c461526612a18cf426	0.43	0.015	0	0	0	0	5.9E-05	5.9E-04	Bacteria	Actinobacteria	Actinobacteria	Actinomycetales	Mycobacteriaceae	*Mycobacterium*	
	675999ae65440500a81da32022f6bbed	0.56	0.02	1.2E-04	1.1E-04	1.4E-04	4.9E-04	2.5E-04	6.6E-04	Bacteria	Actinobacteria	Actinobacteria	Actinomycetales	Mycobacteriaceae	*Mycobacterium*	
	1693b72fb96d3fa45f67065ef3445042	0.69	0.005	0	0	1.2E-04	4.4E-04	1.5E-03	1.4E-03	Bacteria	Actinobacteria	Actinobacteria	Actinomycetales	Nakamurellaceae		
	cbd3bd0c85ddab9285651fccd2878632	0.83	0.005	1.4E-04	1.7E-04	0.0E+00	0	2.1E-03	3.0E-03	Bacteria	Actinobacteria	Actinobacteria	Actinomycetales	Nakamurellaceae		
	cc950e82a489ac26feefd26c69151db0	0.75	0.005	1.8E-04	0	3.6E-05	4.4E-04	6.5E-04	2.3E-03	Bacteria	Actinobacteria	Actinobacteria	Actinomycetales	Nocardioidaceae	*Aeromicrobium*	
	12534616632742aa327b73a7ba944d43	0.62	0.02	2.3E-04	3.4E-04	3.7E-04	2.4E-04	1.0E-03	7.6E-04	Bacteria	Actinobacteria	Actinobacteria	Actinomycetales	Nocardioidaceae	*Kribbella*	
	a77ced46deb83a7d5abc79978e9bda72	0.52	0.02	0	0	0	2.0E-04	2.7E-04	7.8E-04	Bacteria	Actinobacteria	Actinobacteria	Actinomycetales	Nocardioidaceae	*Nocardioides*	*halotolerans*
	b40e445e1ad7edc719d9c2c850533e7c	0.75	0.005	9.8E-05	0	2.3E-04	6.8E-04	9.6E-04	1.1E-03	Bacteria	Actinobacteria	Actinobacteria	Actinomycetales	Nocardioidaceae	*Nocardioides*	
	921589397d2babac632f3404af55d4b2	0.52	0.01	0	0	0	0	1.0E-03	0	Bacteria	Actinobacteria	Actinobacteria	Actinomycetales	Nocardioidaceae	*Nocardioides*	
	156572b928592204f50650c986dd8a9c	0.89	0.005	1.6E-04	1.7E-04	1.6E-04	7.3E-04	1.2E-03	2.7E-03	Bacteria	Actinobacteria	Actinobacteria	Actinomycetales	Nocardioidaceae		
	5a78a8936d287d363796fcce0c138d48	0.75	0.005	3.1E-04	7.5E-04	6.6E-04	6.8E-04	1.2E-03	3.1E-03	Bacteria	Actinobacteria	Actinobacteria	Actinomycetales	Nocardioidaceae		
	a6eac410b54adb67a9dd5b8c4aa0581e	0.56	0.005	0	0	0	2.9E-04	1.6E-04	1.5E-04	Bacteria	Actinobacteria	Actinobacteria	Actinomycetales	Nocardioidaceae		
	b5c5886ff266ea4b0929cabe7b6e8329	0.62	0.005	0	9.2E-04	1.1E-04	0	4.5E-04	3.9E-03	Bacteria	Actinobacteria	Actinobacteria	Actinomycetales	Nocardioidaceae		
	b895b6420568f7b504da11b126b26f3d	0.48	0.01	0	0	0	4.9E-04	2.3E-04	5.4E-04	Bacteria	Actinobacteria	Actinobacteria	Actinomycetales	Nocardioidaceae		
	b9ced9bbc36e66832ca69cbfdf947a3f	0.52	0.015	0	0	0	0	4.5E-04	5.1E-04	Bacteria	Actinobacteria	Actinobacteria	Actinomycetales	Nocardioidaceae		
	49296379921f753e54b7579079833014	0.43	0.025	0	0	0	0	0	3.9E-04	Bacteria	Actinobacteria	Actinobacteria	Actinomycetales	Nocardioidaceae		
	df569f7ed835f82b0655fe40adbab6c8	0.46	0.045	0	2.0E-04	0	0	1.8E-03	0	Bacteria	Actinobacteria	Actinobacteria	Actinomycetales	Nocardioidaceae		
	49a1897b3e8a961a219716df9d1892f5	0.80	0.005	6.1E-04	1.4E-03	7.3E-04	2.4E-03	1.6E-03	3.3E-03	Bacteria	Actinobacteria	Actinobacteria	Actinomycetales	Pseudonocardiaceae	*Actinomycetospora*	
	f7bb60140fc3a1fb648c0f18b8c6d36c	0.58	0.035	2.2E-04	1.1E-03	1.1E-04	2.4E-04	3.3E-04	1.7E-03	Bacteria	Actinobacteria	Actinobacteria	Actinomycetales	Pseudonocardiaceae	*Actinomycetospora*	
	50dfa9590708f5ef6d803812b3d65d3e	0.43	0.05	0	0	0	0	1.2E-04	1.7E-04	Bacteria	Actinobacteria	Actinobacteria	Actinomycetales	Pseudonocardiaceae	*Amycolatopsis*	
	bc5216bcdbfd023b0fad515cd5771d49	0.65	0.005	0	1.4E-04	5.3E-05	7.8E-04	3.7E-04	1.4E-03	Bacteria	Actinobacteria	Actinobacteria	Actinomycetales	Sporichthyaceae	*Sporichthya*	
	4d66ee483deffc2e46ce3c5a29abf062	0.56	0.04	2.5E-04	1.4E-04	1.8E-05	0.0E+00	2.0E-05	1.2E-03	Bacteria	Actinobacteria	Actinobacteria	Actinomycetales			
	f251613ed1b58e9d30a86b2e391bd26e	0.48	0.05	2.0E-05	0	1.4E-04	4.4E-04	3.1E-04	3.4E-04	Bacteria	Actinobacteria	Actinobacteria	Actinomycetales			
	f50cfc8a8ff3bc53c9075487e844ec97	0.37	0.05	0	0	0	0	0	6.1E-04	Bacteria	Actinobacteria	Actinobacteria	Actinomycetales			
	8b336ad5d19e3c1b4dafc3486d4e36ff	0.48	0.01	0	0	0	0	3.1E-04	0	Bacteria	Actinobacteria	Actinobacteria	Micrococcales			
	b439ac260853f8194829b760363386fd	0.48	0.01	0	0	0	2.4E-04	7.8E-05	1.2E-04	Bacteria	Actinobacteria	MB-A2-108	0319-7L14			
	696243d2dedb718b59eb9048cb4e9475	0.74	0.005	7.8E-05	0	1.2E-04	1.5E-03	6.1E-04	1.6E-03	Bacteria	Actinobacteria	Thermoleophilia	Gaiellales	Gaiellaceae		
	748f5e1091680b0ee4428fc49c6ad325	0.79	0.005	5.9E-05	2.2E-04	3.4E-04	4.9E-04	1.5E-03	1.4E-03	Bacteria	Actinobacteria	Thermoleophilia	Gaiellales	Gaiellaceae		
	af2b424aa293a766841b3b8827e25bf7	0.76	0.005	7.8E-05	0	6.0E-04	1.2E-03	5.9E-04	1.6E-03	Bacteria	Actinobacteria	Thermoleophilia	Gaiellales	Gaiellaceae		
	c5d7582acb838506b589e70ec7ebc184	0.56	0.005	0	0	0	0	3.1E-04	2.2E-04	Bacteria	Actinobacteria	Thermoleophilia	Gaiellales	Gaiellaceae		
	f5c0df0c2db923b031c1f4c21c7e5ad8	0.74	0.005	0	0	0	7.8E-04	9.2E-04	9.0E-04	Bacteria	Actinobacteria	Thermoleophilia	Gaiellales	Gaiellaceae		
	0222d3a73fafa53c994878a023dc1efc	0.52	0.01	0	0	0	0	2.2E-04	5.6E-04	Bacteria	Actinobacteria	Thermoleophilia	Gaiellales	Gaiellaceae		
	a0f7e5d96fea8258195ee9e9b6aa42ac	0.43	0.03	0	0	0	0	2.3E-04	1.5E-04	Bacteria	Actinobacteria	Thermoleophilia	Gaiellales	Gaiellaceae		
	30693e16001efdddab455aba23808f99	0.43	0.045	0	0	0	1.5E-04	2.0E-04	0	Bacteria	Actinobacteria	Thermoleophilia	Gaiellales	Gaiellaceae		

Quercus	c07285c15d87efc83a4bd7908eb0a502	0.57	0.015	0	5.6E-05	8.9E-05	2.0E-04	1.8E-04	2.4E-04	Bacteria	Actinobacteria	Thermoleophilia	Solirubrobacterales	Conexibacteraceae		
	94ddce43f30b3de8bd67a4fa1886e95c	0.43	0.015	0	0	0	1.5E-04	2.0E-04	1.7E-04	Bacteria	Actinobacteria	Thermoleophilia	Solirubrobacterales	Patulibacteraceae		
	5254b8ba582220f84dc2e7e53f808d4a	0.43	0.035	0	0	0	9.8E-05	7.8E-05	1.2E-04	Bacteria	Actinobacteria	Thermoleophilia	Solirubrobacterales	Patulibacteraceae		
	2071199791dd6418ab3dc07d50ef4d43	0.52	0.005	0	0	0	0	2.9E-04	1.6E-03	Bacteria	Actinobacteria	Thermoleophilia	Solirubrobacterales			
	9b058dbf4e45edf67a9b1cf325e45f67	0.68	0.005	0	3.1E-04	0	2.5E-03	3.7E-04	1.2E-03	Bacteria	Actinobacteria	Thermoleophilia	Solirubrobacterales			
	b3997a39a26f08d316eb5db73755b147	0.64	0.01	0	1.4E-04	2.7E-04	0	4.1E-04	6.4E-04	Bacteria	Actinobacteria	Thermoleophilia	Solirubrobacterales			
	0cec472e3a2ca3aeabd272364e07e6a6	0.66	0.015	5.9E-05	7.3E-04	2.8E-04	1.5E-03	6.3E-04	9.5E-04	Bacteria	Actinobacteria	Thermoleophilia	Solirubrobacterales			
	dceed9fcfc600d105a2e2190220daf3a	0.37	0.05	0	0	0	3.4E-04	0	9.8E-05	Bacteria	Bacteroidetes	Saprospirae	Saprospirales	Chitinophagaceae	*Flavihumibacter*	
	0fa8f80e7ba892e2ec52157157d86c55	0.67	0.005	0	0	0	1.5E-04	1.4E-04	2.5E-03	Bacteria	Bacteroidetes	Saprospirae	Saprospirales	Chitinophagaceae		
	3676f2751212b17db73ceb4c71ac2264	0.60	0.005	0	0	0	0	8.4E-04	6.8E-04	Bacteria	Bacteroidetes	Saprospirae	Saprospirales	Chitinophagaceae		
	d326bbb64c756840cdf8e484f2ea7652	0.70	0.005	1.6E-04	0	2.0E-04	2.4E-04	1.1E-03	1.7E-03	Bacteria	Bacteroidetes	Saprospirae	Saprospirales	Chitinophagaceae		
	d53e59e92eb37b67e7bd24d82ce5b642	0.67	0.005	0	0	0	4.9E-04	2.5E-04	1.3E-03	Bacteria	Bacteroidetes	Saprospirae	Saprospirales	Chitinophagaceae		
	8b2e4b0d8be925fbdd72f1143f712d16	0.58	0.01	1.2E-04	0	8.9E-05	0	1.8E-04	2.3E-03	Bacteria	Bacteroidetes	Saprospirae	Saprospirales	Chitinophagaceae		
	9220a67cd134bc9946c6bf2efd5677db	0.51	0.01	0	0	3.6E-05	1.5E-04	5.3E-04	0	Bacteria	Bacteroidetes	Saprospirae	Saprospirales	Chitinophagaceae		
	f5970a2d9ba0606eea9bf0c4962c1616	0.58	0.01	1.4E-04	0	1.8E-05	0	3.7E-04	1.1E-03	Bacteria	Bacteroidetes	Saprospirae	Saprospirales	Chitinophagaceae		
	30248d5f67fbffb764be5be30bf8dabe	0.57	0.015	3.9E-05	3.1E-04	1.8E-05	0	1.8E-04	8.3E-04	Bacteria	Bacteroidetes	Saprospirae	Saprospirales	Chitinophagaceae		
	5c68b8908e4dbbc1b106f50084e1f64a	0.48	0.015	0	0	0	0	2.5E-04	4.9E-05	Bacteria	Bacteroidetes	Saprospirae	Saprospirales	Chitinophagaceae		
	4632a52c8a6776053a5576485226d8a4	0.50	0.03	5.9E-05	0	1.8E-05	2.4E-04	2.9E-04	5.9E-04	Bacteria	Bacteroidetes	Saprospirae	Saprospirales	Chitinophagaceae		
	8c4a10c82a9c0e44a6aa0b4633a3ec50	0.54	0.03	0	2.0E-04	3.2E-04	2.0E-04	5.5E-04	4.2E-04	Bacteria	Bacteroidetes	Saprospirae	Saprospirales	Chitinophagaceae		
	b55bfa9e3646deda3866cbc054aaf63a	0.55	0.035	0	2.8E-04	1.2E-04	1.5E-04	1.6E-04	1.5E-03	Bacteria	Bacteroidetes	Saprospirae	Saprospirales	Chitinophagaceae		
	162734b3d02fff9e998aea10c383007e	0.43	0.04	0	0	0	2.0E-04	1.6E-04	4.9E-05	Bacteria	Bacteroidetes	Saprospirae	Saprospirales	Chitinophagaceae		
	6e539678b0c419f24c02c9e9b19fea4f	0.53	0.045	3.9E-05	5.6E-05	1.8E-05	0	1.6E-04	5.1E-04	Bacteria	Bacteroidetes	Saprospirae	Saprospirales	Chitinophagaceae		
	5e301f77b78181286f42e00aed5c6719	0.48	0.015	0	0	0	0	1.4E-04	2.7E-04	Bacteria	Bacteroidetes	Saprospirae	Saprospirales	Saprospiraceae		
	57c82a5103d58ce3d52228d877219d1a	0.52	0.015	0	0	1.8E-05	0	8.0E-04	3.2E-04	Bacteria	Bacteroidetes	Cytophagia	Cytophagales	Cytophagaceae	*Adhaeribacter*	
	bec3ba2dda29e48f28b9db1af76cf968	0.52	0.005	0	0	0	5.9E-04	1.8E-04	3.9E-04	Bacteria	Bacteroidetes	Cytophagia	Cytophagales	Cytophagaceae	*Cytophaga*	
	e8d30f670fe1f65e3b4e69e152700457	0.55	0.015	3.9E-05	5.6E-05	0.0E+00	0	1.8E-04	1.1E-03	Bacteria	Bacteroidetes	Cytophagia	Cytophagales	Cytophagaceae	*Dyadobacter*	
	e613c49262499af6606a34b0c83ff215	0.56	0.005	0	0	0	0	6.3E-04	1.7E-04	Bacteria	Bacteroidetes	Cytophagia	Cytophagales	Cytophagaceae	*Hymenobacter*	
	bfb758aa28184610485715737a961d56	0.52	0.01	0	0	0	0	5.9E-04	8.6E-04	Bacteria	Bacteroidetes	Cytophagia	Cytophagales	Cytophagaceae	*Hymenobacter*	
	ad6b6a41b5b8b1de4bb3bd273ad6e8ee	0.46	0.04	0	0	8.9E-05	0	1.0E-03	0	Bacteria	Bacteroidetes	Cytophagia	Cytophagales	Cytophagaceae	*Hymenobacter*	
	7b18d62fecb8b2a08028e9b01cabecfd	0.43	0.045	0	0	0	0	3.7E-04	0	Bacteria	Bacteroidetes	Cytophagia	Cytophagales	Cytophagaceae	*Hymenobacter*	
	a82bb2bc32ee934173b6757467ff5190	0.46	0.02	0	0	1.8E-05	1.5E-04	7.8E-05	2.0E-04	Bacteria	Bacteroidetes	Cytophagia	Cytophagales	Cytophagaceae	*Spirosoma*	
	37b6d03c6e6080e19857dd3db579c4d9	0.37	0.05	0	0	0	0	0	4.9E-04	Bacteria	Bacteroidetes	Cytophagia	Cytophagales	Cytophagaceae	*Spirosoma*	
	23b870d0574883d71d3be8efbb227f91	0.72	0.005	3.9E-05	2.8E-05	2.0E-04	9.8E-05	4.1E-04	1.3E-03	Bacteria	Bacteroidetes	Cytophagia	Cytophagales	Cytophagaceae		
	19952574bd7aafe954178caa1d093751	0.48	0.015	0	0	0	9.8E-05	9.8E-05	2.4E-04	Bacteria	Bacteroidetes	Cytophagia	Cytophagales	Cytophagaceae		
	d5fd9e1f42e947b4e4317723ba0be0c6	0.48	0.015	0	0	0	4.9E-05	2.0E-05	7.3E-05	Bacteria	Bacteroidetes	Cytophagia	Cytophagales	Cytophagaceae		
	c814f4c589b8e7e84ad7df40c0593f97	0.43	0.03	0	0	0	0	3.5E-04	9.8E-05	Bacteria	Bacteroidetes	Cytophagia	Cytophagales	Cytophagaceae		
	af65e1c188e779e147b11e8ec3613158	0.43	0.035	0	0	0	0	3.9E-05	3.4E-04	Bacteria	Bacteroidetes	Cytophagia	Cytophagales	Cytophagaceae		
	04a465fb9870f8138a525ff38d1ddd52	0.51	0.045	3.9E-05	4.7E-04	5.3E-05	0	1.4E-04	1.7E-03	Bacteria	Bacteroidetes	Cytophagia	Cytophagales	Cytophagaceae		
	e939ef30906bffe172ed90042f19d769	0.52	0.005	0	0	0	4.9E-05	1.8E-04	1.7E-04	Bacteria	Bacteroidetes	Cytophagia	Cytophagales			
	a839f5e596cdfff8069595745b5fc3f0	0.43	0.035	0	0	0	0	1.2E-04	2.9E-04	Bacteria	Bacteroidetes	Flavobacteriia	Flavobacteriales	Cryomorphaceae	*Fluviicola*	
	7d9f90a81cc4cd3f78117b0b87025a54	0.55	0.005	0	0	2.1E-04	6.4E-04	1.7E-03	1.3E-03	Bacteria	Bacteroidetes	Flavobacteriia	Flavobacteriales	Flavobacteriaceae	*Flavobacterium*	*succinicans*
	c9c69005371a5d23290b256b08d5127a	0.70	0.005	0	1.4E-04	0	2.4E-04	3.5E-03	4.9E-03	Bacteria	Bacteroidetes	Flavobacteriia	Flavobacteriales	Flavobacteriaceae	*Flavobacterium*	*succinicans*
	8aa440a88e8c2668f734d21f78ee17ca	0.56	0.005	0	0	0	0	6.8E-04	3.2E-04	Bacteria	Bacteroidetes	Flavobacteriia	Flavobacteriales	Flavobacteriaceae	*Flavobacterium*	
	7ff3c6447d1c3bc8120fd4255dba90ed	0.43	0.03	0	0	0	5.4E-04	7.8E-05	2.2E-04	Bacteria	Bacteroidetes	Flavobacteriia	Flavobacteriales	Flavobacteriaceae	*Flavobacterium*	
	fb5f53c930e182466ac32775e36f8d0b	0.43	0.04	0	0	0	0	4.7E-04	7.3E-05	Bacteria	Bacteroidetes	Flavobacteriia	Flavobacteriales	Flavobacteriaceae	*Flavobacterium*	
	2e1aad0cad41b59e6d162ebd16e51bf5	0.43	0.05	0	0	0	0	5.9E-05	7.6E-04	Bacteria	Bacteroidetes	Flavobacteriia	Flavobacteriales	Flavobacteriaceae	*Flavobacterium*	
	de3ba83cff424d4abc8ad564f27e6ae2	0.47	0.02	0	0	1.8E-05	0	1.8E-04	3.9E-04	Bacteria	Bacteroidetes	Sphingobacteriia	Sphingobacteriales	Chitinopahaceae		
	d93710531847e0d902d724e2e85c7c6c	0.68	0.005	1.8E-04	6.1E-04	0	5.9E-04	5.5E-04	2.2E-03	Bacteria	Bacteroidetes	Sphingobacteriia	Sphingobacteriales	Sphingobacteriaceae		
	e2a5e3151699651d010ed7497072f9dd	0.58	0.015	1.6E-04	0	8.9E-05	9.3E-04	3.5E-04	2.7E-04	Bacteria	Bacteroidetes	Sphingobacteriia	Sphingobacteriales	Sphingobacteriaceae		
	8cc0cb1d08883f00db64a594a4bde667	0.48	0.025	0	0	2.5E-04	2.9E-04	0	1.2E-03	Bacteria	Bacteroidetes	Sphingobacteriia	Sphingobacteriales	Sphingobacteriaceae		
	7676216b4f98baf9d780e56e1f062ce0	0.48	0.015	0	0	0	0	3.9E-05	3.7E-04	Bacteria	Bacteroidetes	Sphingobacteriia	Sphingobacteriales			
	8106981248446e2e29a68c397f018267	0.48	0.02	0	0	0	0	3.3E-04	8.1E-04	Bacteria	Bacteroidetes	Sphingobacteriia	Sphingobacteriales			
	5765b81dbe5bdd5d60a192c3b8606923	0.43	0.04	0	0	0	0	1.2E-04	1.2E-04	Bacteria	Bacteroidetes	Sphingobacteriia	Sphingobacteriales			
	f242b9de35d5a00e13f60fe26810606e	0.43	0.04	0	0	0	0	9.8E-05	2.7E-04	Bacteria	Bacteroidetes	Sphingobacteriia	Sphingobacteriales			
	f44ef27e6fc7b28b690ef48ecd45744d	0.43	0.035	0	0	0	0	9.8E-05	1.7E-04	Bacteria	Chlamydiae	Chlamydiia	Chlamydiales	Parachlamydiaceae		
	da150aff3d59124c280b5fd8472c550e	0.74	0.005	0	0	0	0	4.2E-03	1.4E-03	Bacteria	Chlamydiae	Chlamydiia	Chlamydiales	Rhabdochlamydiaceae	*Candidatus Rhabdochlamydia*	
	92feef56ef65f2f1baa9c42599312672	0.43	0.045	0	0	0	9.8E-05	1.4E-04	2.4E-05	Bacteria	Chloroflexi	Anaerolineae	Caldilineales	Caldilineaceae		
	e77e7ca09de4e691738c41f6d22c6fa7	0.85	0.005	0	0	3.2E-04	1.2E-03	1.2E-03	2.1E-03	Bacteria	Chloroflexi	Ellin6529				
	961f2becef6912795fe0c1cc16e12a31	0.58	0.015	0	1.1E-04	8.9E-05	6.4E-04	8.0E-04	6.4E-04	Bacteria	Chloroflexi	Ellin6529				
	71af765feffee8202dffa36591fe3933	0.48	0.015	0	0	0	0	2.5E-04	3.2E-04	Bacteria	Chloroflexi	Thermomicrobia	JG30-KF-CM45			
	e33daa9a783bd93fb95d82f30e5e0662	0.61	0.015	0	0	2.8E-04	4.9E-05	9.8E-05	7.8E-04	Bacteria	Chloroflexi	Thermomicrobia	JG30-KF-CM45			
	b246f4a628aeeddc94bab124f9bfa95a	0.43	0.045	0	0	0	0	7.8E-05	3.7E-04	Bacteria	Chloroflexi	TK10				
	8c00569991341a9c5d6968a5581d70f2	0.65	0.01	7.8E-05	1.1E-04	5.3E-05	2.0E-04	3.9E-04	6.4E-04	Bacteria	FBP					
	e344eff9c512c8663b55a3717f2e4ece	0.57	0.015	5.9E-05	0	1.2E-04	9.3E-04	5.9E-05	9.5E-04	Bacteria	Firmicutes	Bacilli	Bacillales			
	a6e0b6890c4fb18dcd7c7d89d4ec05c3	0.37	0.05	0	0	0	0	0	1.1E-03	Bacteria	Gemmatimonadetes	Gemm-3				
	5124885f63e9dd60a82c8eb40a9d632a	0.73	0.005	0	2.8E-05	5.3E-05	9.8E-05	3.7E-04	1.8E-03	Bacteria	Gemmatimonadetes	Gemmatimonadetes	Ellin5290			
	69c6663304a146545f3af78aeed799d5	0.53	0.04	1.2E-04	2.8E-05	3.6E-05	0	2.2E-04	8.6E-04	Bacteria	Gemmatimonadetes	Gemmatimonadetes	Gemmatimonadales	Ellin5301		
	52023db770e05c849d0e1991ee9e61c4	0.55	0.005	2.0E-05	0	0	1.5E-04	1.2E-04	2.2E-04	Bacteria	Planctomycetes	Phycisphaerae	WD2101			
	8aa91a9bb2e12936b82df2be7bb696f3	0.43	0.015	0	0	0	0	2.2E-04	1.7E-04	Bacteria	Planctomycetes	Phycisphaerae	WD2101			
	94b1cc19ae9e10f36c3a5bcbfe341735	0.48	0.03	0	0	0	0	0	7.1E-04	Bacteria	Planctomycetes	Planctomycetia	Gemmatales	Gemmataceae	*Gemmata*	
	4ac60229e9f4bfe058026f231e41c94d	0.43	0.025	0	0	0	0	3.5E-04	4.9E-05	Bacteria	Planctomycetes	Planctomycetia	Gemmatales	Isosphaeraceae		
	b6be1fcc7523d2b58f9fa80874bed789	0.56	0.01	0	0	0	0	3.9E-05	5.6E-04	Bacteria	Planctomycetes	Planctomycetia	Pirellulales	Pirellulaceae		
	124a556a7ea65bd973b47bf28634a1ea	0.51	0.025	9.8E-05	0	0	0	2.5E-04	1.5E-04	Bacteria	Planctomycetes	Planctomycetia	Pirellulales	Pirellulaceae		
	d89f1df6b9aa75464d404a6565a3e27a	0.51	0.045	5.9E-05	0	1.1E-04	0	7.8E-05	7.1E-04	Bacteria	Planctomycetes	vadinHA49	DH61			
	e7da441a22df372da317ba29a23b241b	0.43	0.035	0	0	0	3.4E-04	2.5E-04	4.6E-04	Bacteria	Proteobacteria	Alphaproteobacteria	Caulobacterales	Caulobacteraceae	*Phenylobacterium*	
	3bd21c1f2b1ae487f30791218b8444c0	0.43	0.045	0	0	0	0	3.5E-04	5.1E-04	Bacteria	Proteobacteria	Alphaproteobacteria	Caulobacterales	Caulobacteraceae	*Phenylobacterium*	
	2ab277d01012b4877e312134efe376ef	0.48	0.01	0	0	0	0	9.8E-05	5.9E-04	Bacteria	Proteobacteria	Alphaproteobacteria	Caulobacterales	Caulobacteraceae		
	3240bbdd4b69ecec44545fd2d79b8349	0.65	0.005	1.2E-04	0	4.1E-04	0	5.3E-04	2.1E-03	Bacteria	Proteobacteria	Alphaproteobacteria	Ellin329			
	a1f35be34e60d2f8a0f191506107e7aa	0.43	0.04	0	0	0	2.9E-04	4.1E-04	0	Bacteria	Proteobacteria	Alphaproteobacteria	Rhizobiales	Beijerinckiaceae		
	e9c41661de978067f9dc87f31a1346aa	0.43	0.045	0	0	0	0	1.2E-04	4.9E-04	Bacteria	Proteobacteria	Alphaproteobacteria	Rhizobiales	Beijerinckiaceae		
	19f50e066230463f30a9423a2f0541e0	0.62	0.035	4.9E-04	3.9E-04	5.3E-05	0	2.8E-03	5.9E-04	Bacteria	Proteobacteria	Alphaproteobacteria	Rhizobiales	Bradyrhizobiaceae	*Bosea*	*genosp.*
	93242c9bd125296f580cec1290db2745	0.43	0.02	0	0	0	0	1.6E-04	9.8E-05	Bacteria	Proteobacteria	Alphaproteobacteria	Rhizobiales	Bradyrhizobiaceae		
	bf981d491f31ae07e725d9af65538c34	0.71	0.005	0	0	3.2E-04	2.5E-03	8.6E-04	1.8E-03	Bacteria	Proteobacteria	Alphaproteobacteria	Rhizobiales	Hyphomicrobiaceae	*Rhodoplanes*	
	114159617d8501c001b0fee1fac85cf3	0.48	0.01	0	0	0	2.4E-04	1.8E-04	9.8E-05	Bacteria	Proteobacteria	Alphaproteobacteria	Rhizobiales	Hyphomicrobiaceae	*Rhodoplanes*	
	6c310ab85bb0a16cabcbd78e66152de5	0.61	0.01	1.6E-04	0	1.6E-04	4.9E-04	3.7E-04	7.6E-04	Bacteria	Proteobacteria	Alphaproteobacteria	Rhizobiales	Hyphomicrobiaceae	*Rhodoplanes*	
	6598429447ed4906911ed1b36f9c17ca	0.48	0.015	0	0	0	0	7.2E-04	6.4E-04	Bacteria	Proteobacteria	Alphaproteobacteria	Rhizobiales	Hyphomicrobiaceae	*Rhodoplanes*	
	a9a07cbb160266ff09cf49ad5d8b8c9a	0.52	0.015	1.2E-04	0	3.4E-04	0	7.4E-04	5.1E-04	Bacteria	Proteobacteria	Alphaproteobacteria	Rhizobiales	Hyphomicrobiaceae	*Rhodoplanes*	
	50df0c9c9204b507957b249b495cfb3e	0.49	0.03	0	4.2E-04	0	3.9E-04	2.3E-04	1.6E-03	Bacteria	Proteobacteria	Alphaproteobacteria	Rhizobiales	Hyphomicrobiaceae	*Rhodoplanes*	
	9c6fba5073178ddcfceb388ba50e74bf	0.55	0.04	2.5E-04	0	5.7E-04	4.4E-04	5.5E-04	7.8E-04	Bacteria	Proteobacteria	Alphaproteobacteria	Rhizobiales	Hyphomicrobiaceae	*Rhodoplanes*	
	3c93aac1890ad3b6d555dd39fecf43a3	0.43	0.05	0	0	0	0	9.8E-05	1.7E-04	Bacteria	Proteobacteria	Alphaproteobacteria	Rhizobiales	Methylocystaceae		
	2bcc7082f4a29b154bcaa87cf00b4fe3	0.65	0.005	0	0	8.9E-05	2.0E-04	6.3E-04	3.2E-04	Bacteria	Proteobacteria	Alphaproteobacteria	Rhizobiales			
	c074a2274e5729d65eb0663d1f173fb8	0.67	0.005	0	0	0	2.0E-04	8.4E-04	1.5E-03	Bacteria	Proteobacteria	Alphaproteobacteria	Rhizobiales			
	f7a30b939c4a85d3c2092dda6c4fd1da	0.45	0.05	3.9E-05	0	0	0	9.8E-05	2.4E-04	Bacteria	Proteobacteria	Alphaproteobacteria	Rhodobacterales	Hyphomonadaceae		
	20b5d39b025cc1c5567fd1a4188ad92a	0.43	0.04	0	0	0	9.8E-05	0	3.4E-04	Bacteria	Proteobacteria	Alphaproteobacteria	Rhodobacterales	Rhodobacteraceae	*Amaricoccus*	
	16357c104069b5c62302e913480dce46	0.48	0.01	0	0	0	0	3.9E-05	7.1E-04	Bacteria	Proteobacteria	Alphaproteobacteria	Rhodobacterales	Rhodobacteraceae	*Rhodobacter*	
	3ddf979d19b34e87c800066f689dd4de	0.56	0.005	0	0	0	2.0E-04	1.8E-04	3.2E-04	Bacteria	Proteobacteria	Alphaproteobacteria	Rhodospirillales	Acetobacteraceae		
	96eaed9254d060e7b3d9ae95817d8675	0.46	0.045	0	0	5.3E-05	0	2.9E-04	3.9E-04	Bacteria	Proteobacteria	Alphaproteobacteria	Rhodospirillales	Acetobacteraceae		
	34f1a1134a2f5433fd6b90de56403842	0.43	0.045	0	0	0	4.9E-05	1.4E-04	4.9E-04	Bacteria	Proteobacteria	Alphaproteobacteria	Rhodospirillales	Rhodospirillaceae	*Reyranella*	*massiliensis*
	edc7320c36b321d26066a36bd73675b6	0.68	0.01	0	1.7E-04	2.7E-04	1.5E-04	2.3E-04	2.0E-03	Bacteria	Proteobacteria	Alphaproteobacteria	Rhodospirillales	Rhodospirillaceae		
	8c1333e1a10dbe084e3d5c682f508030	0.43	0.025	0	0	0	0	2.0E-05	2.2E-04	Bacteria	Proteobacteria	Alphaproteobacteria	Rhodospirillales	Rhodospirillaceae		
	ad46db2c47310296436d04a4828225f0	0.43	0.035	0	0	0	0	5.1E-04	7.1E-04	Bacteria	Proteobacteria	Alphaproteobacteria	Rhodospirillales	Rhodospirillaceae		
	4f6a1817209eea1406f51edc45e63813	0.53	0.02	3.9E-05	0	7.1E-05	0	2.5E-04	6.1E-04	Bacteria	Proteobacteria	Alphaproteobacteria	Sphingomonadales	Erythrobacteraceae		
	be112bb54f52e77f3836018ae718d8e8	0.61	0.035	9.8E-05	1.7E-04	8.7E-04	2.4E-04	1.0E-03	1.2E-03	Bacteria	Proteobacteria	Alphaproteobacteria	Sphingomonadales	Erythrobacteraceae		
	947bb131c525264f69000efd5ad1fd83	0.50	0.005	5.9E-05	0	0	2.4E-04	4.3E-04	1.5E-04	Bacteria	Proteobacteria	Alphaproteobacteria	Sphingomonadales	Sphingomonadaceae	*Kaistobacter*	
	c5c846de8bbeb794e2d6f11d08210274	0.48	0.02	0	0	0	7.8E-04	1.4E-04	0	Bacteria	Proteobacteria	Alphaproteobacteria	Sphingomonadales	Sphingomonadaceae	*Kaistobacter*	
	a5a10ae51d2c30edf6d2f6aa53d077bf	0.43	0.04	0	0	0	0	2.3E-04	1.2E-04	Bacteria	Proteobacteria	Alphaproteobacteria	Sphingomonadales	Sphingomonadaceae	*Kaistobacter*	
	4e0bd3f74934f144c2f7fb894a914daa	0.61	0.025	2.9E-04	3.4E-04	0	0	6.3E-04	7.3E-04	Bacteria	Proteobacteria	Alphaproteobacteria	Sphingomonadales	Sphingomonadaceae	*Novosphingobium*	
	fabbe13c47cfae3d114f6fdc31ba8390	0.43	0.04	0	0	0	0	2.9E-04	0	Bacteria	Proteobacteria	Alphaproteobacteria	Sphingomonadales	Sphingomonadaceae	*Novosphingobium*	

	d03e0a7fdacf9904b6e657921bb1308b	0.48	0.03	0	0	0	0	2.0E-05	1.2E-03	Bacteria	Proteobacteria	Alphaproteobacteria	Sphingomonadales	Sphingomonadaceae	*Sphingobium*	
	4c9ad6211096cef7339b99102a7ab037	0.48	0.045	0	1.7E-04	2.1E-04	4.9E-04	5.7E-04	0	Bacteria	Proteobacteria	Alphaproteobacteria	Sphingomonadales	Sphingomonadaceae	*Sphingomonas*	*wittichii*
	5cb573c9685b3a7612abd1da56bff581	0.64	0.005	0	0	0	0	2.7E-04	4.2E-04	Bacteria	Proteobacteria	Alphaproteobacteria	Sphingomonadales	Sphingomonadaceae	*Sphingomonas*	
	5c6a716d746ce6913292730ede2a4fff	0.55	0.01	0	1.4E-04	0	0	1.4E-03	0	Bacteria	Proteobacteria	Alphaproteobacteria	Sphingomonadales	Sphingomonadaceae	*Sphingomonas*	
	c8d0fc260798ab91ac26b30fe4ec6ae2	0.48	0.015	0	0	0	3.4E-04	2.2E-04	1.5E-04	Bacteria	Proteobacteria	Alphaproteobacteria	Sphingomonadales	Sphingomonadaceae	*Sphingomonas*	
	07bb4dc1f9ec324a60d78a51cbdc9e0f	0.56	0.005	0	0	0	2.0E-04	4.5E-04	2.4E-04	Bacteria	Proteobacteria	Alphaproteobacteria	Sphingomonadales	Sphingomonadaceae		
	d865b5425239838757e91a4bf8e2832a	0.55	0.045	2.5E-04	1.7E-04	2.5E-04	2.9E-04	5.1E-04	1.0E-03	Bacteria	Proteobacteria	Alphaproteobacteria	Sphingomonadales	Sphingomonadaceae		
	86610b92c6fe08140453eea21ac2f677	0.52	0.01	0	3.1E-04	0	4.4E-04	8.8E-04	0	Bacteria	Proteobacteria	Betaproteobacteria	Burkholderiales	Burkholderiaceae	*Burkholderia*	*andropogonis*
	38a438c0ac980ee192378b94dd688973	0.56	0.005	0	0	0	4.4E-04	6.8E-04	3.2E-04	Bacteria	Proteobacteria	Betaproteobacteria	Burkholderiales	Comamonadaceae		
	6d2d187f44fa7f751e8ca227b27335e6	0.74	0.005	0	4.5E-04	0	3.9E-04	1.0E-03	1.9E-03	Bacteria	Proteobacteria	Betaproteobacteria	Burkholderiales	Comamonadaceae		
	3f0b0108af3848e5b83c520c3fc0da88	0.62	0.015	4.1E-04	1.7E-04	1.2E-04	4.4E-04	9.0E-04	8.8E-04	Bacteria	Proteobacteria	Betaproteobacteria	Burkholderiales	Comamonadaceae		
	d9d980e45e7118c91301ed3c168b9af7	0.63	0.02	0	3.7E-03	2.3E-04	0	7.6E-04	9.3E-03	Bacteria	Proteobacteria	Betaproteobacteria	Burkholderiales	Comamonadaceae		
	58623e4dbeb0df6dbddd0e1ab2212f3c	0.47	0.02	0	8.4E-05	0	0	6.5E-04	4.6E-04	Bacteria	Proteobacteria	Betaproteobacteria	Burkholderiales	Oxalobacteraceae	*Janthinobacterium*	
	b57744bd30af9f7a4d538d0aba27596b	0.52	0.04	0	8.9E-04	2.3E-04	9.8E-05	3.4E-03	7.8E-04	Bacteria	Proteobacteria	Betaproteobacteria	Burkholderiales	Oxalobacteraceae	*Janthinobacterium*	
	20ad5b343a158e617aa2f39adbf005b6	0.72	0.01	5.7E-04	1.4E-04	7.6E-04	4.9E-04	1.3E-03	3.0E-03	Bacteria	Proteobacteria	Betaproteobacteria	Burkholderiales	Oxalobacteraceae		
	a07502d409329c1fbb54d1c037cfd53c	0.48	0.005	0	0	0	0	1.4E-04	8.6E-04	Bacteria	Proteobacteria	Betaproteobacteria	Ellin6067			
	8fb04b080ed1f9936da6e8d3f2be08d0	0.90	0.005	0	4.2E-04	0	1.0E-03	1.2E-03	2.2E-03	Bacteria	Proteobacteria	Betaproteobacteria	IS-44			
	909f3b5ca78551ccc9e0ece36da14a62	0.51	0.01	0	1.4E-04	0	0	2.2E-04	1.2E-03	Bacteria	Proteobacteria	Betaproteobacteria	Methylophilales	Methylophilaceae		
	fa2d8337da84d098aade0b59d0cc4172	0.48	0.015	0	0	0	0	9.8E-05	5.9E-04	Bacteria	Proteobacteria	Betaproteobacteria	Methylophilales			
	913e48d9e2caae8fdb73d2822a52a26b	0.43	0.035	0	0	0	9.8E-05	0	2.0E-04	Bacteria	Proteobacteria	Betaproteobacteria	MND1			
	e348dd0f8b01220a2d4fc152bc8feb4e	0.48	0.01	0	0	0	2.9E-04	2.5E-04	4.9E-05	Bacteria	Proteobacteria	Betaproteobacteria	SC-I-84			
	7a00364cedfd47ae39cc0e46cecaf831	0.46	0.015	2.0E-05	0	0	9.8E-05	5.9E-05	2.0E-04	Bacteria	Proteobacteria	Betaproteobacteria	SC-I-84			
	ad4db7a139d3fd19f38897fa9dee062f	0.49	0.015	9.8E-05	0	1.8E-05	2.4E-04	5.5E-04	1.7E-04	Bacteria	Proteobacteria	Betaproteobacteria	SC-I-84			
	81b750e461c4c19b71bb69a7be144baf	0.59	0.035	7.8E-05	5.6E-05	6.9E-04	0	7.8E-04	1.0E-03	Bacteria	Proteobacteria	Betaproteobacteria	SC-I-84			
	f344f26cf00db9133c7fa5cc072bbad6	0.46	0.04	0	0	3.6E-05	0	3.5E-04	2.0E-04	Bacteria	Proteobacteria	Betaproteobacteria	SC-I-84			
	280baf6980eaaba7af3824b1f2d6f695	0.43	0.045	0	0	0	2.0E-04	0	2.0E-04	Bacteria	Proteobacteria	Betaproteobacteria	SC-I-84			
	06f249e121d0aa52f0a23d83a77224e5	0.72	0.01	0	3.9E-04	1.0E-03	7.8E-04	1.2E-03	1.6E-03	Bacteria	Proteobacteria	Betaproteobacteria				
	2ef6e4e62813fdf7c2f8edc5bfc59a1f	0.43	0.025	0	0	0	9.8E-05	5.9E-05	2.4E-05	Bacteria	Proteobacteria	Betaproteobacteria				
	71a54a61b54175de9f11706f40f0b5f8	0.43	0.05	0	0	0	2.4E-04	7.8E-05	2.4E-05	Bacteria	Proteobacteria	Deltaproteobacteria	Bdellovibrionales	Bdellovibrionaceae	*Bdellovibrio*	
	80f2c70db5de14b0d28d768354709385	0.69	0.005	1.4E-04	0	0	2.9E-04	3.9E-04	1.5E-03	Bacteria	Proteobacteria	Deltaproteobacteria	Myxococcales	Haliangiaceae		
	ada110d0cf8e1a306a122f30a9d43f37	0.43	0.035	0	0	0	0	0	1.8E-03	Bacteria	Proteobacteria	Deltaproteobacteria	Myxococcales	Haliangiaceae		
	9dbe74d9725508672f27037bb7a1fb63	0.43	0.05	0	0	0	0	2.2E-04	1.5E-04	Bacteria	Proteobacteria	Deltaproteobacteria	Myxococcales	Nannocystaceae	*Nannocystis*	
	34bd5a925c1c9bbd21ed956019c25e71	0.48	0.03	0	0	0	9.8E-05	3.5E-04	5.6E-04	Bacteria	Proteobacteria	Deltaproteobacteria	Myxococcales	Polyangiaceae	*Aetherobacter*	*fasciculatus*
	77b5b85096e0946b8dc42af546197489	0.43	0.045	0	0	0	9.8E-05	0	7.6E-04	Bacteria	Proteobacteria	Deltaproteobacteria	Myxococcales	Polyangiaceae		
	4c28f04149785f51fed32e961cd0f86f	0.64	0.005	3.9E-05	2.8E-05	1.2E-04	9.8E-05	9.4E-04	4.4E-04	Bacteria	Proteobacteria	Deltaproteobacteria	Myxococcales			
	cc86356cdd74156181e6812fcb472deb	0.52	0.005	0	0	0	0	2.7E-04	2.0E-04	Bacteria	Proteobacteria	Deltaproteobacteria	Myxococcales			
	d741925b8c69408b61996dd11534f374	0.60	0.005	0	1.1E-04	1.2E-04	0	2.0E-04	1.2E-03	Bacteria	Proteobacteria	Deltaproteobacteria	Myxococcales			
	f862421f10ce11ec0b8b77a54a4cdf44	0.73	0.005	2.3E-04	0	1.2E-04	3.4E-04	7.4E-04	1.9E-03	Bacteria	Proteobacteria	Deltaproteobacteria	Myxococcales			
	82d69a8fa3757238ead7808bb0d1b068	0.48	0.01	0	0	0	4.9E-04	0	5.1E-04	Bacteria	Proteobacteria	Deltaproteobacteria	Myxococcales			
	ca7edd2c0c85a7b9898a8e641b457e50	0.48	0.015	0	0	0	0	7.8E-05	4.9E-04	Bacteria	Proteobacteria	Deltaproteobacteria	Myxococcales			
	59e5a05f7e45e5518c4ae40d05a8f19c	0.43	0.025	0	0	0	0	0	5.9E-04	Bacteria	Proteobacteria	Deltaproteobacteria	Myxococcales			
	078da005b9b0a34902761d6bdcfdabe5	0.43	0.03	0	0	0	0	2.0E-05	2.0E-04	Bacteria	Proteobacteria	Deltaproteobacteria	Myxococcales			
	72f9c77f01a4cadceceebca8280affef	0.43	0.03	0	0	0	2.9E-04	5.9E-05	0	Bacteria	Proteobacteria	Deltaproteobacteria	Myxococcales			
	ca85b434502aa8e8c97d97cc9ec81843	0.53	0.05	0	0	3.6E-04	4.9E-05	3.1E-04	4.2E-04	Bacteria	Proteobacteria	Deltaproteobacteria	Myxococcales			
	c9e7926bf66bf2f8d7ed26a705d76e58	0.52	0.01	0	0	0	2.4E-04	2.2E-04	1.2E-04	Bacteria	Proteobacteria	Deltaproteobacteria	Syntrophobacterales	Syntrophobacteraceae		
	622d4b277eb8055836e26b649058428b	0.48	0.025	0	0	0	0	0	1.1E-03	Bacteria	Proteobacteria	Gammaproteobacteria	Alteromonadales	211ds20		
	1688f14bc9ca0ccc2aeae650cd6e6553	0.56	0.005	0	2.8E-05	0	2.0E-04	7.8E-05	9.3E-04	Bacteria	Proteobacteria	Gammaproteobacteria	Alteromonadales	OM60		
	0e37606c473a0d3e570c379897bae26a	0.43	0.03	0	0	0	9.8E-05	5.9E-05	4.9E-05	Bacteria	Proteobacteria	Gammaproteobacteria	HTCC2188	HTCC2089		
	e27680d4009f98f30248d823bc17fb8e	0.37	0.045	0	0	0	1.4E-02	2.0E-05	0	Bacteria	Proteobacteria	Gammaproteobacteria	Pasteurellales	Pasteurellaceae	*Haemophilus*	*parainfluenzae*
	f8e023e914fb9e5e3950dadf8e7f177f	0.58	0.015	2.0E-05	0	5.3E-05	4.4E-04	1.4E-04	6.1E-04	Bacteria	Proteobacteria	Gammaproteobacteria	Pseudomonadales	Moraxellaceae		
	d269a2365f5e0c51faad3d4c14e17278	0.49	0.035	0	0	2.1E-04	9.8E-04	8.4E-04	0	Bacteria	Proteobacteria	Gammaproteobacteria	Pseudomonadales	Pseudomonadaceae	*Pseudomonas*	
	cc635e9ce849498d61436833850e1459	0.54	0.015	0	2.2E-04	0	0	2.0E-04	3.9E-04	Bacteria	Proteobacteria	Gammaproteobacteria	Xanthomonadales	Sinobacteraceae	*Alkanibacter*	*difficilis*
	3558bc5e3381a3f199cf592d72c83055	0.43	0.035	0	0	0	2.9E-04	0	2.7E-04	Bacteria	Proteobacteria	Gammaproteobacteria	Xanthomonadales	Sinobacteraceae	*Panacagrimonas*	*perspica*
	8460d749c54a4dd2cde6dd7c7388d686	0.51	0.01	2.0E-05	0	0	4.9E-05	2.0E-04	9.8E-05	Bacteria	Proteobacteria	Gammaproteobacteria	Xanthomonadales	Sinobacteraceae		
	8ed314126fe22cbf3f45f64e859ff87e	0.52	0.01	0	0	0	1.5E-04	2.9E-04	4.9E-05	Bacteria	Proteobacteria	Gammaproteobacteria	Xanthomonadales	Sinobacteraceae		
	d0f57ae2a67f063bd64cf5500a14e066	0.62	0.01	2.2E-04	8.4E-05	3.9E-04	2.0E-04	2.3E-04	1.8E-03	Bacteria	Proteobacteria	Gammaproteobacteria	Xanthomonadales	Sinobacteraceae		
	c7d56c84b33907b6662ffd6d3491fe5b	0.52	0.015	0	0	0	0	3.9E-05	7.3E-04	Bacteria	Proteobacteria	Gammaproteobacteria	Xanthomonadales	Sinobacteraceae		
	d3d9ff9c5a32af82c53a2922fd8a009e	0.58	0.015	0	0	1.2E-04	0	1.0E-03	7.8E-04	Bacteria	Proteobacteria	Gammaproteobacteria	Xanthomonadales	Sinobacteraceae		
	b021e462c13785b8b7a719136ff3f0e8	0.60	0.045	0	3.1E-04	3.7E-04	0	1.2E-04	2.1E-03	Bacteria	Proteobacteria	Gammaproteobacteria	Xanthomonadales	Sinobacteraceae		
	461f8f7cd00ec2cc29bf94bd013c266b	0.43	0.045	0	0	0	0	3.5E-04	5.1E-04	Bacteria	Proteobacteria	Gammaproteobacteria	Xanthomonadales	Xanthomonadaceae	*Pseudoxanthomonas*	
	b757da9aab56247367c4be7bb9cd0a8a	0.48	0.015	0	0	0	4.9E-05	2.3E-04	1.2E-04	Bacteria	Proteobacteria	Gammaproteobacteria	Xanthomonadales	Xanthomonadaceae	*Thermomonas*	
	b94769eb72718a0c512ade94384e3902	0.89	0.005	1.4E-04	2.2E-04	4.4E-04	1.0E-03	3.1E-03	1.2E-03	Bacteria	Proteobacteria	Gammaproteobacteria	Xanthomonadales	Xanthomonadaceae		
	03af51e75c7ee46771685036b7c12f28	0.48	0.01	0	0	0	5.4E-04	9.8E-05	6.4E-04	Bacteria	Proteobacteria	Gammaproteobacteria	Xanthomonadales	Xanthomonadaceae		
	c68c29d09b76c6738c7e138485cb0638	0.80	0.005	0	0	1.2E-03	6.8E-04	2.1E-03	3.6E-03	Bacteria	Proteobacteria					
	9d8d2207f5cd5cc728ecaf66d15806d7	0.37	0.05	0	0	0	0	0	2.0E-04	Bacteria	Proteobacteria					
	35aadd30ee9d0eb6978b890cbe2dc733	0.43	0.05	0	0	0	0	2.0E-04	1.2E-04	Bacteria	Verrucomicrobia	Methylacidiphilae	Methylacidiphilales			
	e21c06957a2167fcfe5f32907679ae37	0.52	0.01	0	0	0	0	0	9.0E-04	Bacteria	Verrucomicrobia	Pedosphaerae	Pedosphaerales	auto67_4W		
	385b00f19a2598a2de1b9e467a26d923	0.48	0.005	0	0	0	1.5E-04	1.2E-04	5.9E-04	Bacteria	Verrucomicrobia	Pedosphaerae	Pedosphaerales			
	271b576cc1f2ce84b35d8374ed909db4	0.75	0.005	0	0	1.2E-03	1.6E-03	1.1E-03	2.2E-03	Bacteria	Verrucomicrobia	Spartobacteria	Chthoniobacterales	Chthoniobacteraceae	*DA101*	
	9bb1f6bce7dfedc12951e62ee0ff2b6f	0.65	0.005	0	0	7.1E-05	7.3E-04	2.3E-04	5.9E-04	Bacteria	Verrucomicrobia	Spartobacteria	Chthoniobacterales	Chthoniobacteraceae	*DA101*	
	57f3562d588b974e7979619da315d1f6	0.52	0.01	0	0	0	5.9E-04	2.2E-04	9.8E-05	Bacteria	Verrucomicrobia	Spartobacteria	Chthoniobacterales	Chthoniobacteraceae	*Ellin506*	
	9bcdfc8204acdd298aba85b062603595	0.57	0.02	0	8.4E-05	1.6E-04	0	3.9E-05	1.7E-03	Bacteria	Verrucomicrobia	Spartobacteria	Chthoniobacterales	Chthoniobacteraceae	*Ellin506*	
	734e4849488a1c091a492e8f2ea94cd0	0.64	0.005	0	0	0	9.8E-05	0	1.5E-03	Bacteria	Verrucomicrobia	Opitutae	Opitutales	Opitutaceae	*Opitutus*	
	450b51af1ad7e19a21c38edd56d3794d	0.43	0.035	0	0	0	3.9E-04	3.5E-04	2.0E-04	Bacteria	Verrucomicrobia	Opitutae	Opitutales	Opitutaceae	*Opitutus*	
	7fe72824f63885bb1dd42b9e81723164	0.47	0.01	0	0	1.8E-05	0	0	8.8E-04	Bacteria	Verrucomicrobia	Opitutae	Opitutales	Opitutaceae		
	102ef5be319f433b5bf1ff9c0c80658f	0.54	0.03	0	0	6.4E-04	4.9E-05	6.3E-04	1.8E-03	Bacteria	Verrucomicrobia	Opitutae	Opitutales	Opitutaceae		
	22d0ce457b2606ee94ce5ead360574bc	0.72	0.005	1.6E-04	1.1E-04	3.6E-05	1.5E-04	7.4E-04	1.0E-03	Bacteria	Verrucomicrobia	Verrucomicrobiae	Verrucomicrobiales	Verrucomicrobiaceae	*Luteolibacter*	
	9635838b901fef669bfc1cea54093b6d	0.58	0.05	0	5.6E-04	2.0E-04	4.9E-05	3.7E-04	1.3E-03	Bacteria	Verrucomicrobia	Verrucomicrobiae	Verrucomicrobiales	Verrucomicrobiaceae	*Luteolibacter*	
	d7000205ff0d985c4491a0907fdb8e39	0.43	0.015	0	0	0	0	0	3.7E-04	Bacteria	Verrucomicrobia					
	149c761c4dad186ed1a111f29292f7bf	0.48	0.02	0	0	0	0	3.9E-05	2.2E-04	Bacteria						
