# Supplementary material for: Invasive vegetation affects amphibian skin microbiota and body condition
Source: PeerJ. 2020 Feb 19;8:e8549. doi: 10.7717/peerj.8549 (PMC7035873; doi:10.7717/peerj.8549)
Supplement: Supplemental Information 1 [file peerj-08-8549-s001.zip › PeerJ Supp/supplemental_tables_captions.docx]

Supplemental Table Captions

Table S1: *Batrachoseps attenuatus* microhabitat soil microbiota indicator species analysis results. ASVs associated with either *Eucalyptus* or *Quercus* habitat soils are presented. For each ASV the LDA statistic (effect size), p-value, average relative abundance at each site, and taxonomy is presented. Relative abundance values are colored based on value from 0 (white) to 0.1 (black).

Table S2: *Batrachoseps attenuatus* skin microbiota indicator species analysis results. ASVs associated with salamanders presiding in either *Eucalyptus* or *Quercus* habitat are presented. For each ASV the LDA statistic (effect size), p-value, average relative abundance at each site, and taxonomy is presented. Relative abundance values are colored based on value from 0 (white) to 0.1 (black).
